# Omnidirectional 3D Printing of PEDOT: PSS Aerogels with Tunable Electromechanical Performance: A Playground for Unconventional Stretchable Interconnects and Thermoelectrics

**DOI:** 10.1002/advs.202412491

**Published:** 2025-01-22

**Authors:** Hasan Emre Baysal, Tzu‐Yi Yu, Viktor Naenen, Stijn De Smedt, Defne Hiz, Bokai Zhang, Heyi Xia, Isidro Florenciano, Martin Rosenthal, Ruth Cardinaels, Francisco Molina‐Lopez

**Affiliations:** ^1^ Department of Materials Engineering KU Leuven Kasteelpark Arenberg 44 Leuven 3001 Belgium; ^2^ Department of Chemical Engineering, Soft Matter, Rheology and Technology (SMaRT) KU Leuven Celestijnenlaan 200J Leuven 3001 Belgium; ^3^ Department of Chemistry KU Leuven Celestijnenlaan 200f Leuven 3001 Belgium

**Keywords:** organic thermoelectrics, PEDOT:PSS, printed electronics, stretchable electronics, thermoelectric aerogels

## Abstract

The next generation of soft electronics will expand to the third dimension. This will require the integration of mechanically compliant 3D functional structures with stretchable materials. Here, omnidirectional direct ink writing (DIW) of poly(3,4‐ethylenedioxythiophene): polystyrene sulfonate (PEDOT:PSS) aerogels with tunable electrical and mechanical performance is demonstrated, which can be integrated with soft substrates. Several PEDOT:PSS hydrogels are formulated for DIW and freeze‐dried directly on stretchable substrates to form integrated aerogels displaying high shape fidelity and minimal shrinkage. This technology demonstrates 3D‐structured stretchable interconnects, planar thermoelectric generators for skin electronics, and vertically printed high aspect ratio thermoelectric pillars with ultralow thermal conductivity of 0.065 W m^−1^ K^−1^. The aerogel pillars outpower their dense counterparts in realistic energy harvesting scenarios, where contact resistances cannot be ignored and produced up to 26 nW cm^−2^ (corresponding to a gravimetric power density of 0.76 mW kg^−1^) for a difference of temperature of 15 K. Here, promising advancements in soft and energy‐efficiency electronic systems relevant to soft robotics and wearables are suggested.

## Graphical abstract

Here, 3D‐printable poly(3,4‐ethylenedioxythiophene): polystyrene sulfonate aerogels that are both conductive and mechanically robust, along with a novel protocol for integrating them directly onto stretchable substrates are presented. Omnidirectional direct ink writing enables the production of complex shapes. The technology's versatility is showcased through stretchable interconnects and thermoelectric generators, suggesting potential applications in soft robotics and wearable power sources.

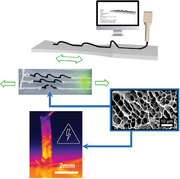

## 1 Introduction

Standing at the intersection of electrical engineering and materials science, soft electronics refers to a burgeoning class of electronic systems that share the form factor of biological systems,^[^ ^1^ ^]^ i.e., they are mechanically soft and stretchable and can take up complex 3D shapes. Soft electronics offer tremendous potential for application in soft robotics,^[^ ^2^ ^]^ wearable electronics,^[^ ^3^, ^4^, ^5^, ^6^ ^]^ biomedical devices,^[^ ^4^, ^7^ ^]^ and tissue engineering.^[^ ^8^ ^]^ The steep development of the soft electronics field comes with enhanced functionalities and an ever‐increasing energy demand that batteries cannot always fulfill either because they are too bulky or rigid for specific applications or because regular replacement might be inconvenient, unfeasible, or environmentally unsustainable. Hence, powering soft electronics requires versatile renewable power sources with compatible unconventional form factors like three‐dimensionality and deformability.

Thermoelectric materials can generate electrical power from heat flows. Compared to competing harvesting technologies such as photovoltaics, piezoelectrics, or triboelectrics,^[^ ^6^ ^]^ thermoelectric (TEs) do not require light, mechanical vibrations, or friction to operate, making them ideal candidates to power wearables by exploiting the temperature gradient between the human body and the surrounding environment. The performance of a TE material is evaluated by the dimensionless figure of merit *ZT*, defined as *ZT = σS^2^T/κ*, where *σ, S, T*, and *κ* denote electrical conductivity, Seebeck coefficient, absolute temperature, and thermal conductivity, respectively. The Seebeck coefficient measures a material's ability to generate an electric voltage in response to a temperature gradient. The power factor (PF* = σ S^2^*) indicates the effectiveness of a material in producing electricity from a temperature difference. The power factor is often used to evaluate TE materials when *κ* is inaccessible^[^ ^9^ ^]^ or when net power generation prevails over efficiency.^[^ ^10^ ^]^ The realm of TE materials operating at room temperature is dominated by the bismuth telluride (Bi_2_Te_3_) family. Despite their indisputable top performance (*ZT* > 1)^[^ ^11^ ^]^ and the recent advances toward the production of large‐area, flexible, and printed Bi_2_Te_3_‐based devices compatible with wearables,^[^ ^12^, ^13^, ^14^, ^15^, ^16^ ^]^ their widespread use is limited by their reliance on expensive elements with a relatively high environmental impact and geographically constrained availability.

Alternatively, organic thermoelectric materials—often conducting polymers—are abundant, can be produced sustainably, are easy to process from solution, and are intrinsically soft. Those attributes make them a perfect alternative to Bi_2_Te_3_ for room‐temperature applications where performance is not the main factor to consider. Poly(3,4‐ethylenedioxythiophene): polystyrene sulfonate (PEDOT:PSS) is one of the most popular thermoelectric polymers due to its superior ambient stability and relatively high performance.^[^ ^17^ ^]^ PEDOT:PSS thin films displayed the record high performance among organic TEs, boasting an impressive *ZT* > 0.25 and PF > 500 µW m^−1^ K^−1^).^[^ ^18^, ^19^ ^]^ The key to the high TE performance of PEDOT:PSS resides in its high electrical conductivity. Moreover, its mechanical properties and water stability can be improved by adding ionic salts that act as plasticizers^[^ ^20^, ^21^, ^22^, ^23^ ^]^ and PSS crosslinkers like (3‐glycidyloxypropyl)trimethoxysilane (GOPS).^[^ ^24^, ^25^ ^]^ Despite the undoubtful potential of PEDOT:PSS for organic TEs, the most impressive values reported for PF and *ZT* correspond to thin films below 1 µm, which are unfortunately of little use for practical thermoelectrics as they lead to high internal resistance and low output power (Equation (1)).^[^ ^15^ ^]^ Attempts to make thicker films result irremediably in much lower performance than the found in thin‐film counterparts (500–800 S cm^−1[^ ^26^, ^27^ ^]^ vs 1000–2500 S cm^−1^).^[^ ^27^, ^28^ ^]^ Moreover, TE generators fabricated with thin films are constrained to planar geometries that do not adapt optimally to most real‐life scenarios, in which the hot and cold surfaces are opposed to each other and the heat travels perpendicular to the substrate surface.^[^ ^14^ ^]^

Planar form factors do not fulfill the shape versatility required by emerging soft electronics either. Unlike traditional electronics, soft circuits are not exclusively planar. Hence, they require fabrication strategies that move beyond thin‐film techniques and toward the third dimension,^[^ ^29^, ^30^, ^31^, ^32^, ^33^ ^]^ a requirement that 3D printing fulfills. In particular, direct ink writing (DIW) allows additive manufacturing of intricate self‐supported 3D structures by extruding ink through a nozzle directly on a substrate.^[^ ^30^ ^]^ DIW of soft electronic materials such as conjugated polymers^[^ ^30^, ^34^ ^]^ and hybrid organic–inorganic composites^[^ ^32^ ^]^ has been reported for applications such as tissue engineering,^[^ ^8^ ^]^ soft robotics,^[^ ^30^ ^]^ electromagnetic shielding,^[^ ^35^ ^]^ and 3D electrical interconnects.^[^ ^33^, ^36^ ^]^ DIW has also shown potential for inorganic TEs,^[^ ^37^ ^]^ but it has seldom been reported for organic TEs.^[^ ^38^ ^]^ The few 3D‐structured organic TE devices with a high aspect ratio that have been reported involved complex processing like filling prepatterned wells^[^ ^39^ ^]^ or painting preshaped legs.^[^ ^40^ ^]^ DIW of organic TE materials remains elusive for the following reasons. First, developing conducting polymer inks with the required rheology for DIW, i.e., shear thinning and high yield stress,^[^ ^30^, ^41^ ^]^ without jeopardizing their electrical performance, is challenging. Recent efforts in the field demonstrated routes to formulate DIW‐printable PEDOT:PSS inks with decent conductivities of tens of S cm^−1^. Those routes include blending PEDOT:PSS with insulating phases, such as hydrogels or nonvolatile liquids,^[^ ^42^ ^]^ and promoting electrostatic interaction between polymer chains.^[^ ^36^, ^42^, ^43^, ^44^ ^]^ Second, substantial volume shrinkage and loss of shape fidelity occur in DIW parts upon solvent drying, thereby defeating the main purpose of 3D printing. Some authors proposed freeze‐drying or supercritical‐drying of hydrogels as a successful route to avoid shrinkage, resulting in porous and lightweight aerogels.^[^ ^45^, ^46^, ^47^ ^]^ Interestingly, PEDOT:PSS aerogels present extremely low thermal conductivity, a desired trait for thermoelectric applications.^[^ ^45^, ^46^, ^47^ ^]^ However, this decrease in thermal conductivity comes at the expense of an even more substantial reduction in electrical conductivity, resulting in an overall decrease in the figure of merit, *ZT*. A priori, this trade‐off makes aerogels uninteresting for TE devices. Additionally, researchers have shown that PEDOT:PSS aerogels can be formulated to be mechanically robust and flexible.^[^ ^48^, ^49^ ^]^ However, 3D printing of stretchable PEDOT aerogels is yet to be demonstrated.

Despite the great recent progress in DIW PEDOT:PSS, its direct integration into a device remains a challenge.^[^ ^42^ ^]^ In the case of printed hydrogels, shrinkage upon solvent drying leads to shape loss and interfacial stress with the substrate, which weakens adhesion. In the case of printed aerogels, shape retention is ensured, but weak adhesion remains an issue because the abrupt temperature difference involved in freeze‐ and supercritical drying builds up thermal stress at the substrate interface and promotes delamination. This reliability situation worsens for stretchable substrates, where mechanical stress during operation adds to interfacial stresses built up during fabrication. Indeed, no demonstration exists of the direct integration of a PEDOT:PSS aerogel on a substrate (rigid or stretchable) to produce a device. Instead, the reported devices based on PEDOT:PSS aerogels rely on the fabrication of standalone aerogels that must be incorporated manually into a device in a nonscalable manner.

**Figure 1:**
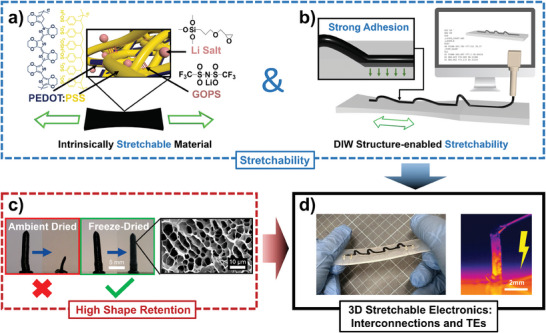
Overview of the materials, processing, and integration for 3D stretchable electronics. a) Intrinsically stretchable PEDOT:PSS is achieved by incorporating GOPS (crosslinker) and Li salt (plasticizer). The interaction between the PEDOT:PSS and additives is schematically illustrated. b) Digital fabrication of PEDOT:PSS out‐of‐plane structures by DIW provide additional stretchability to the design. c) Freeze‐drying ensures high shape retention postprinting. Optical photograph displaying the shrinkage difference between ambient drying and freeze‐drying for 3D‐printed pillars. Scanning electron microscopy (SEM) image shows the porous structure of the freeze‐dried aerogel. d) The proposed technology introduces a playground for 3D stretchable electronics and thermoelectrics (TEs).

In this study, we report omnidirectional DIW of highly conductive and stretchable PEDOT:PSS aerogel parts with high shape fidelity and demonstrate, for the first time, its direct integration into stretchable substrates (**Figure** **1**). Carefully formulated pastes, based on Li salt and GOPS additives, allow tailoring the balance between electrical conductivity and mechanical stretchability to a particular application (Figure 1a). For those applications requiring stretchability, such as stretchable interconnects and planar TE generators suitable for skin electronics, the intrinsic stretchability of the PEDOT:PSS is complemented by the structure‐based stretchability endowed by the free‐forming capability of DIW and by the synergistic integration with a stretchable substrate (Figure 1b). Simultaneously, a high shape retention of the printed parts is ensured by freeze‐drying to form stable aerogels (Figure 1c). This technology introduces a playground for soft and freeform electronics. To illustrate this concept, stretchable interconnects were demonstrated where the maximum strain could be tuned by material formulation and design: in arched lines, the maximum strain increased at 5%/arch. Those interconnects were repurposed as one of the legs of a stretchable planar thermocouple that could find application as a skin‐mounted generator or self‐powered thermometer (Figure 1d). Both devices showed strain insensitivity up to 10% in an unoptimized device. Furthermore, DIW was used to fabricate pillar‐like organic TE legs with a high aspect ratio of up to ≈7 (Figure 1d). Such a TE generator configuration is desirable for exploiting the ubiquitous through‐plane thermal gradients available in our surroundings, increasing the applicability of TE energy harvesters for real‐life applications. Our printed pillars achieved a *ZT *= 3.2 × 10^−3^. Although this performance is low compared to thin film PEDOT:PSS, it is comparable with the best‐reported organic TE aerogels (Table S6, Supporting Information). Most importantly, the high porosity of the aerogel resulted in an ultralow thermal conductivity of 0.065 W m^−1^ K^−1^, which led to higher thermal gradients across the aerogel pillars than across dense pillars with comparable geometry. Surprisingly, the aerogel pillars also supplied more electrical power than their dense counterparts despite using ≈90% less material. We show that this counterintuitive result originates from the unavoidable contact resistances in real devices. The 3D‐printed pillars yielded a high thermoelectric power generation of 26 nW cm^−2^ under conditions mimicking a wearable device attached to the skin and operating indoors (Δ*T* = 15 K and no heat sink placed at the cold side). This areal power density value is among the highest reported so far for skin‐mounted organic TE harvesters and for organic TEs in general. The lightweight and material reduction enabled by the aerogel phase yielded a high gravimetric power density of 0.76 mW kg^−1^ (at Δ*T* = 15 K). This parameter, overlooked so far in the field, is paramount for the viability of portable and low‐cost devices. The simultaneous high TE performance, mechanical flexibility, and light weight of our 3D‐printable organic TE aerogels, along with their compatible integration on stretchable substrates, represent a new playground for the fabrication of unconventional stretchable interconnects and self‐powered soft electronic systems for the next generation of soft robots and wearables.

## 2 Results and Discussion

### 2.1 Formulation and Processing

PEDOT:PSS is one of the most performing and ambient‐stable p‐type organic thermoelectric materials and these properties have been underpinned by extensive literature. PEDOT:PSS comprises a conjugated conducting phase, PEDOT, and an insulating counter polyanion PSS. Common commercial formulations of PEDOT:PSS include an excess of hydrophilic PSS in the blend that facilitates the dispersibility of the hydrophobic PEDOT phase in water. However, this excess of PSS hinders the formation of a densely connected network of conductive PEDOT. Instead, it leads to isolated “pancake‐like” PEDOT islands with low crystallinity that endow the material with low electrical conductivity (0.2–1 S cm^−1^).^[^ ^17^ ^]^ One of the most typical ways to increase the electrical conductivity of pristine PEDOT:PSS is by adding the so‐called secondary dopants, i.e., polar solvents such as dimethyl sulfoxide (DMSO) and ethylene glycol (EG), which promotes a morphology change from the “pancake‐like” morphology to connected and highly crystalline PEDOT fibers.^[^ ^19^, ^50^ ^]^ The electrical conductivity can be further improved by removing the excess insulating PSS via postprocessing washing steps.^[^ ^51^, ^52^ ^]^ Preprocessing has recently been proposed to remove the excess PSS: vacuum‐assisted filtration of a commercial PEDOT:PSS aqueous dispersion diluted in EG resulted in a high conductivity of around 2500 S cm^−1^.^[^ ^28^ ^]^ Some secondary dopants for PEDOT:PSS, such as ionic liquids and nonionic polymeric surfactants, like Triton X‐100 or Zonyl, not only increase the electrical conductivity but also act as plasticizers to promote mechanical stretchability.^[^ ^20^, ^21^, ^22^, ^23^ ^]^ Other additives such as GOPS^[^ ^24^, ^25^ ^]^ and 4‐dodecylbenzenesulfonic acid^[^ ^53^ ^]^ enhance the mechanical properties and water stability by inducing crosslinking of the PSS or the PEDOT phase, respectively.

**Figure 2:**
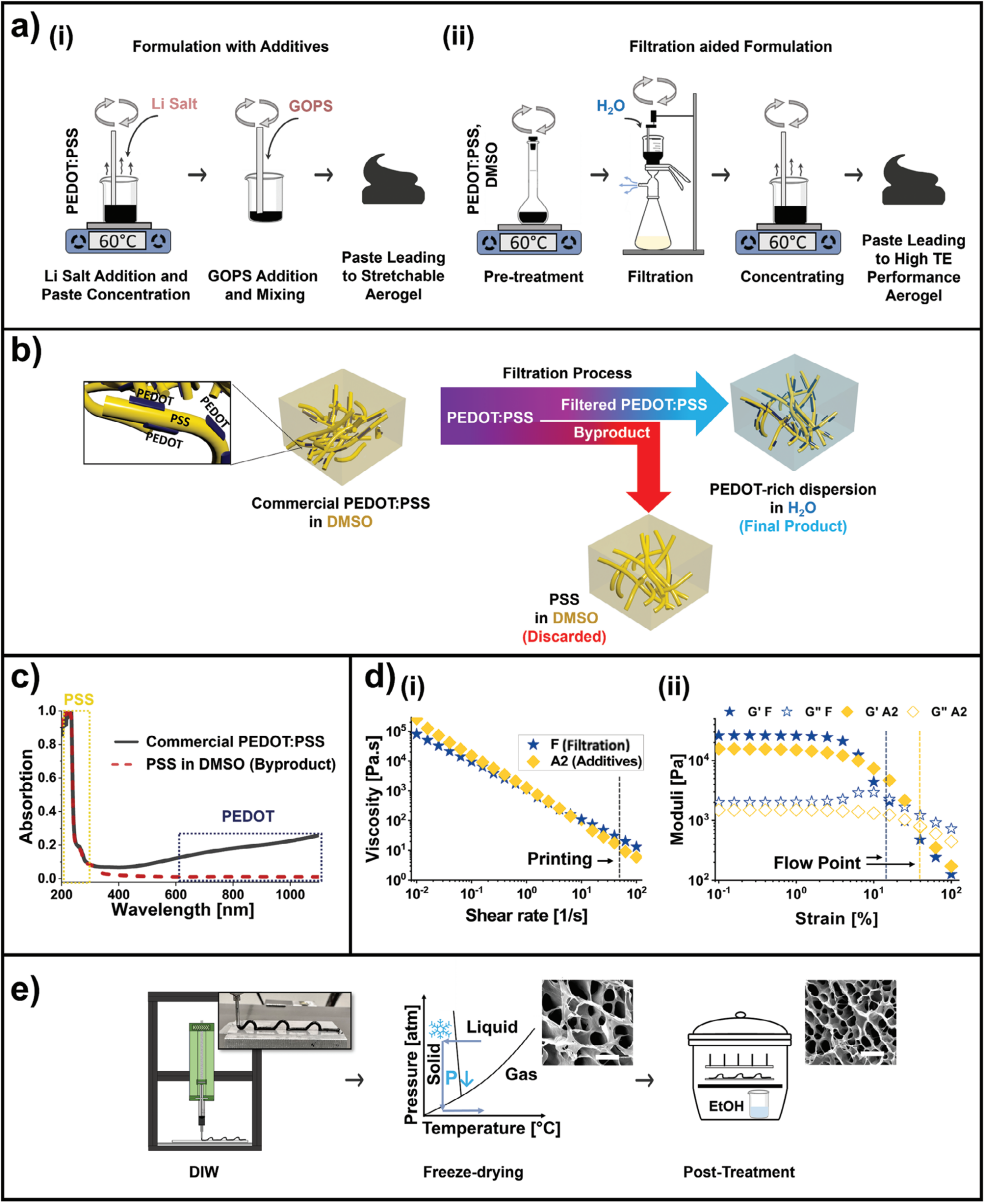
Process flow of 3D‐printed aerogel‐based stretchable organic thermoelectrics. a,i) Process flow for paste leading to high‐stretchability aerogels, which involves formulation with additives: addition of Li salt into PEDOT:PSS, concentration, and addition of GOPS and ii) process flow for aerogels with a high thermoelectric performance, which involves filtration‐aided formulation: mixing of PEDOT:PSS with the dimethyl sulfoxide (DMSO) secondary dopant, vacuum‐assisted filtration to remove excess of PSS, DMSO‐water solvent exchange (under continuous mixing), and concentration. b) Schematic representation of the effect of the filtration process in the paste morphology showing the discarded byproduct and the final product consisting of a PEDOT‐rich PEDOT:PSS viscous dispersion in water. c) UV–vis absorption spectrum of the commercial PEDOT:PSS dispersion in DMSO before filtration and the discarded byproduct containing PSS. d) Rheology of the concentrated printable pastes formulated with additives (*A2*) and assisted by filtration (*F*), including i) the viscosity versus shear rate flow curve and ii) strain sweep. e) The process flow of paste preparation, including direct ink writing (DIW) of the paste, freeze‐drying, and post‐treatment with ethanol vapor. Optical photograph of representative 3D‐printed arches. Scanning electron micrographs (SEM) of the aerogel (from the *F* hydrogel) before and after ethanol vapor post‐treatment (scale bars correspond to 10 µm).

Therefore, a dispersion of off‐the‐shelf high‐conductivity PEDOT:PSS was used as a benchmark material to prepare 3D‐printable aerogel‐based organic thermoelectrics compatible with a stretchable substrate. Various formulations were evaluated. However, as detailed in the subsequent discussion, no combination yielded optimal thermoelectric and mechanical performance simultaneously. Nevertheless, our findings revealed that a wide range of electromechanical properties can be covered by two distinct process routes: one involving additives and the other incorporating DMSO excess, which is subsequently removed via filtration‐assisted solvent (water‐DMSO) exchange. The former provided superior mechanical properties, and the latter the best thermoelectric performance. These two different types of paste preparation steps are illustrated in **Figure** **2**a. For the preparation of the pastes with additives (suitable for stretchable electronics applications, Figure 2ai), the PEDOT:PSS dispersion was mixed with bis(trifluoromethane)sulfonimide lithium salt (called Li salt in the rest of the paper). The dispersion was transferred in an open Teflon container, where it was partially dried at 60 °C while continuously stirring until a hydrogel with a suitable viscosity for DIW was achieved. This corresponded to 1.42 g paste per 10 mL of the original PEDOT:PSS dispersion.^[^ ^54^ ^]^ When the correct viscosity was reached, the paste was removed from the hotplate, and the crosslinker GOPS was added. The crosslinker was added to the hydrogel in the last step of the paste preparation to avoid crosslinking before printing. Li salt increased the stretchability^[^ ^21^ ^]^ of the final material by acting as a plasticizer. It develops soft regions within the polymer (amorphous part), vital for attaining a high failure strain.^[^ ^21^ ^]^ Simultaneously, Li salt improved the conductivity by diminishing the electrostatic interactions between PEDOT and PSS, thereby enabling PEDOT to form a “hard” conductive network with high connectivity embedded within the more pliable PSS matrix.^[^ ^21^ ^]^ Excessive Li salt, however, can cause instability due to salt leaching. To counteract this issue and reinforce the material, GOPS is used to induce crosslinking within the PSS (Figure 1a), thereby stabilizing the overall structure, stabilizing the salt inside the PEDOT:PSS structure, and strengthening the mechanical properties of the final material.^[^ ^24^, ^55^ ^]^

To prepare the pastes with filtration for high‐performance TE applications (Figure 2aii), the PEDOT:PSS dispersion was mixed with DMSO for three days at 60 °C in a close vial. DMSO is a typical secondary dopant for PEDOT:PSS^[^ ^17^, ^22^ ^]^ that promotes a morphology transition from a low‐conductivity structure consisting of PSS‐isolated PEDOT islands to high‐conducting and well‐connected PEDOT fibers.^[^ ^19^, ^27^ ^]^ This morphology transition occurs in dispersion,^[^ ^19^, ^27^ ^]^ and once it happens, the DMSO can be washed away to recover a greener water‐based system (Figure 2b). DMSO must also be removed because it acts as an antifreezing agent, impeding the freeze‐drying step planned downstream in the process flow. On the other hand, the PEDOT:PSS dispersion used in this study contains 2.5 times more insulating PSS than conducting PEDOT, which hinders conductivity. The DMSO‐to‐water solvent exchange and the removal of excess PSS were achieved in a modified vacuum filtration system (Figure 2ai) by filtering away the excess PSS from the PEDOT:PSS‐DMSO mixture with the help of iterative water washing. The result was a PSS‐rich DMSO/water solution to be discarded at the bottom of the setup and a PEDOT‐rich aqueous dispersion on top of the filter (Figure 2b). The efficient removal of excess PSS was confirmed by the UV‐visible light spectrum of the filtered‐out byproduct (Figure 2c), which displayed absorption features exclusively attributed to PSS (peak positioned ≈200 nm).^[^ ^56^ ^]^ As a comparison, the original PEDOT:PSS dispersion showed both absorption features of PSS and PEDOT (band from 500 to 1200 nm,^[^ ^57^ ^]^ spectra normalized to the PSS feature). The removal of the PSS is also supported by a change in the morphology evidenced by SEM and atomic force microscope (AFM) (Figure S1, Supporting Information). In agreement with a previous study,^[^ ^28^ ^]^ the excess of PSS in pristine PEDOT smooths out the granular texture otherwise encountered in the filtration‐assisted material. Figure 4 provides further insight into the structure of the different aerogels. The PEDOT‐rich dispersion remaining at the top of the filter was then transferred to an open Teflon container, where it was partially dried at 60 °C while continuously stirring until forming a hydrogel with the suitable viscosity for DIW.

To verify whether the rheology of the hydrogels is suitable for DIW, flow curves; frequency, strain, stress sweep tests, and creep‐recovery tests were conducted (Figure 2d and Figure S2, Supporting Information). The flow curves (viscosity vs shear rate, Figure 2di and Figure S2a, Supporting Information) confirm a desired low viscosity of ≈50 Pa s at the printing shear rate, roughly estimated at around 30 s^−1^ (Equations (S1) and (S2), Supporting Information). This low viscosity ensures flow in the nozzle. The flow curve data correspond to a yield stress plateau of ≈1000 Pa (Figure S2b, Supporting Information). This yield stress is more than sufficient to overcome retraction of the printed filament under the influence of surface tension, with the Laplace pressure being ≈175 Pa for the used nozzle size (Equation (S5), Supporting Information). Moreover, due to the high yield stress, the hydrogel can sustain the gravitational stress of extra deposited material on top, allowing the printing of vertical pillars up to an estimated height of 0.2 m with good shape retention (Equation (S6), Supporting Information). The frequency sweep test shows the gel (elastic) character of the inks under low deformations (*G*′ > *G*″, Figure S1c, Supporting Information). The strain sweep test (Figure 2dii and Figure S2d, Supporting Information) demonstrates that the materials exhibit dominantly elastic behavior up to a relatively large strain of around 10%, at which the flow point is reached. Interestingly, a creep‐recovery test at a stress value below the yield stress unveiled a limited elastic recovery when the shear stress was removed (Figure S2e, Supporting Information). This low elastic recovery is desirable to prevent shapes that were extruded (under strain) from “bouncing” back to their original shape. Furthermore, significant viscous creep below yield stress was only observed for the reference formulation (hydrogel *R*). Significant viscous creep is to be avoided as it can lead to paste flow, jeopardizing the shape fidelity of 3D‐printed structures. Finally, our improved ink formulations contributed a higher yield stress value than the reference hydrogel (Figure S2b,f, Supporting Information). The rheology study shows that the change in the morphology induced by additives or removal of excess PSS takes place in the dispersion. This hypothesis, supported by small angle X‐ray scattering (SAXS) studies,^[^ ^19^, ^27^ ^]^ is here corroborated by the rheology for the first time.

The rheology of the hydrogel allowed omnidirectional printing to form complex shapes such as out‐of‐plane arches (Figure 2e, Movies S1 and S2, Supporting Information) and high aspect ratio (≈7) vertical pillars (Figure 1d) produced directly on a silicone stretchable substrate. This approach offers enhanced processing flexibility compared to conventional layer‐by‐layer deposition methods. However, similar to typical hydrogel printing processes, the printed material experiences dramatic and unpredictable shrinkage, impairing shape retention and leading to surface delamination.^[^ ^42^ ^]^ To counteract the shrinkage, the printed materials were immersed in liquid nitrogen and subjected to freeze‐drying to produce stable aerogels (Figures 1c and 2e). To increase the attachment between the printed material and the stretchable substrate and prevent substrate delamination due to the thermal stresses induced during freeze‐drying (Movie S3, Supporting Information), a thin film of semicured silicone was deposited on the substrate to serve as an adhesion layer. The porous aerogel structure (Figures 1d and 2e) offers advantages in terms of reduced material usage, lightweight, and low thermal conductivity, which are crucial for thermoelectric applications. However, the porosity also leads to a reduction in electrical conductivity. The printed aerogels were subjected to ethanol vapor treatment to mitigate this issue partially. This post‐treatment respected the original porosity of the material, improved its surface by welding loose edges (as depicted in Figure 2e and Figure S3, Supporting Information), and led to a significant boost in electrical conductivity (Figures S5a and S7a, Supporting Information). Although dipping in acid and polar solvents are popular post‐treatments to increase the conductivity of PEDOT:PSS,^[^ ^17^ ^]^ such wet processes are not an option in this case because the capillarity tension induced by drying liquids collapse the aerogel pores and lead to shrinkage and loss of shape (Figure S4, Supporting Information).^[^ ^58^ ^]^

### 2.2 Aerogel Characterization

**Figure 3:**
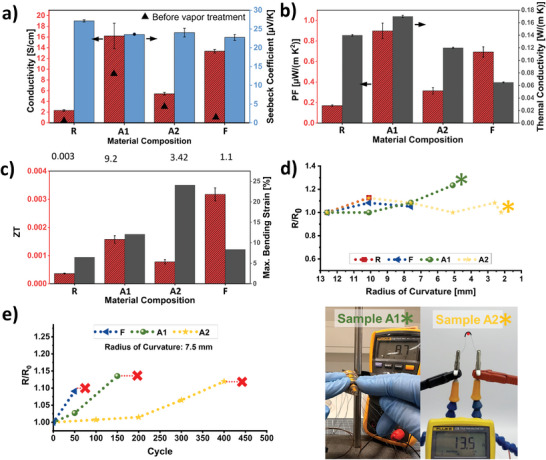
Thermoelectrical and mechanical characterization of 3D printed aerogel filaments to create a library of performing materials. a) Electrical conductivity and Seebeck coefficient. The triangles show the electrical conductivity before ethanol vapor treatment. b) Power factor and thermal conductivity. c) Figure of merit (*ZT*) and maximum survived bending strain. d) The electrical resistance changes of printed aerogels upon bending around a different radius of curvature and optical photograph of the utilized setup. e) The electrical resistance changes in a cyclic bending test around a 7.5 mm radius of curvature (*R* sample is excluded because it did not survive a single bending event at that radius). All samples were post‐treated via ethanol solvent annealing and characterized at ambient conditions. (*R, F, A1*, and *A2* refer to reference PEDOT:PSS, filtration‐assisted PEDOT:PSS, and compositions with Li salt plus low and high concentrations of GOPS, respectively, as described in Figure S5a and Table S1, Supporting Information).

**Figure 4:**
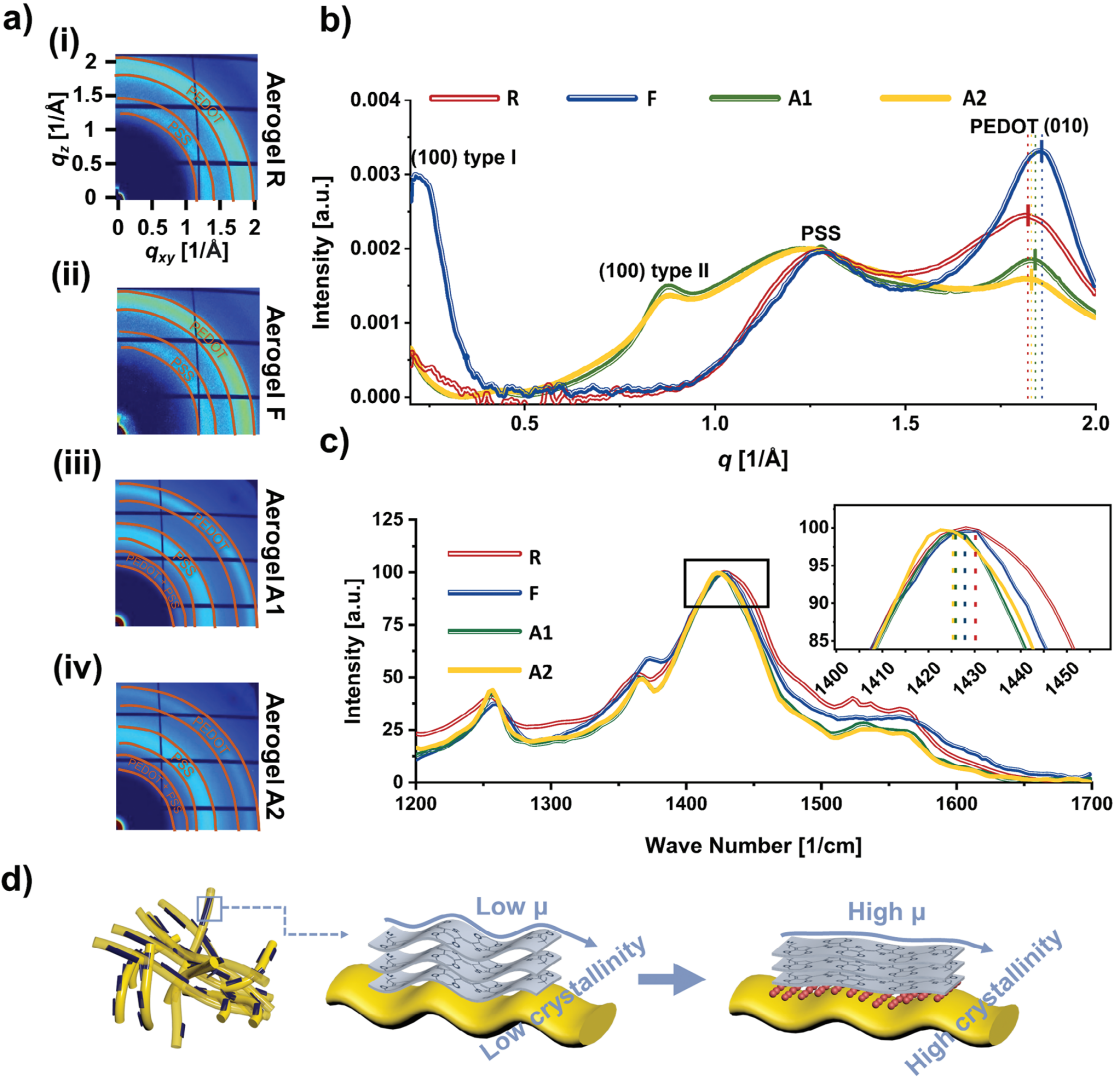
Material morphological characterization of the aerogels made from the selected compositions. a) Synchrotron wide‐angle X‐ray scattering (WAXS) 2D patterns of aerogels i) *R*, ii) *F*, iii) *A1*, and iv) *A2*. b) WAX line‐cut intensity profile normalized to PSS peak intensity. c) Raman spectra and close view inset of the main peak shift. d) Schematic illustration of morphology change in PEDOT:PSS after adding Li salt (*µ* = mobility). (*R*, *F*, *A1*, and *A2* refer to reference PEDOT:PSS, filtration‐assisted PEDOT:PSS, and compositions with Li salt plus low and high concentrations of GOPS, respectively, as described in Figure S5a and Table S1, Supporting Information).

Prior to producing aerogels, thick films were used to evaluate different material compositions (Table S1 and Figure S5a, Supporting Information), and we preselected the most promising materials based on their thermoelectric (Figure S5, Supporting Information) and mechanical (Figure S6, Supporting Information) properties. The compositions selected were the filtration‐assisted PEDOT:PSS (*F*), two compositions with different amounts of GOPS but the same amount of Li salt (*A1* and *A2*, see Table S1 and Figure S5a, Supporting Information). Finally, pristine PEDOT:PSS was used as a reference (*R*). The electrical conductivity and the Seebeck coefficient of the preselected aerogel compositions are shown in **Figure** **3**a. The aerogel samples displayed the same trends across the different material compositions as their thick film counterparts (Figure S8, Supporting Information). In all cases, ethanol vapor post‐treatment boosted the electrical conductivity. Both sets featured a similar Seebeck coefficient (slightly higher for aerogels). However, the aerogels’ conductivity was about 20 times lower than this of dense films, only 16 ± 2 S cm^−1^ for the most conducting aerogel formulation *A1*. This substantial decrease in electrical conductivity was expected, considering the large amount of air in the aerogel (≈90% vol). The high air volume fraction introduces substantial “dead volume,” disrupting conductive pathways and increasing resistance. This results in lower conductivity than the denser thick films, where the PEDOT:PSS network is more interconnected. To counteract this effect, we focused on enhancing the conductivity of the PEDOT:PSS via processes compatible with the formation of DIW aerogels with high shape retention. Filtration‐assisted pretreatment was one of these techniques: the initial PEDOT:PSS aqueous dispersion was diluted in DMSO and subjected to the filtration process described in Figure 2aii,b before freeze‐drying. The filtration aimed to promote highly conducting PEDOT fibrous morphology^[^ ^19^, ^27^ ^]^ and remove the excess insulating PSS from the paste. The filtration process successfully boosted the electrical conductivity of pristine PEDOT:PSS aerogels from 2.3 ± 0.2 to 13.4 ± 0.3 S cm^−1^ (sample *F*). However, filtration did not improve the performance of those formulations containing additives (Figure S8, Supporting Information). This is why the filtered versions of *A1* and *A2* (*1.1f* and *3.1f*, respectively) were not considered for further study. In the end, power factors as high as 0.89 µW m^−1^ K^−2^ and 0.69 µW m^−1^ K^−2^ were achieved for aerogels *A1* and *F*, respectively (Figure 3b). The evolution of the aerogels’ electrical conductivity and Seebeck coefficient with temperature (from −10 to 90 °C) was also investigated and is shown in Figure S9 in the Supporting Information. Interestingly, conductivity increased with temperature for sample *R* but was flat for *F* and decreased for *A1* and *A2*, suggesting a transition from a charge hopping‐dominated transport for *F* to metallic‐like transport for *A1* and *A2*.^[^ ^59^ ^]^ This transition has been attributed to an increase in crystallinity,^[^ ^51^, ^60^ ^]^ which we also encountered and discussed here (**Figure** **4**). The Seebeck coefficient followed a characteristic u‐shaped curve with the minimum inconveniently located around room temperature. To the best of the authors’ knowledge, this u‐shaped behavior has never been reported.

Furthermore, the filtration step reduced the already‐low thermal conductivity of the PEDOT:PSS aerogel *R* (*κ* = 0.1397 ± 0.0013 W m^−1^ K^−1^) to an extremely low value of *κ* = 0.0651 ± 0.0008 W m^−1^ K^−1^ (Figure 3b). This observation suggests that PSS is strongly involved in heat conduction. Indeed, aerogel *F* showed a lower thermal diffusivity, *α*, than *R* (Table S3, Supporting Information, note that similar density, *ρ*, and specific heat capacity, *C*_p_, values were measured for both aerogels and that *κ = α∙ρ∙C*_p_). Such low thermal conductivity of aerogel *F* led to the highest figure of merit *ZT = T∙*PF*/κ* = (3.2 ± 0.2) × 10^−3^ of all the aerogel formulations tested (Figure 3c). This value is among the highest reported for organic TE aerogels (Table S6, Supporting Information). The samples with additives also displayed a lower thermal diffusivity than the reference (*R*). Still, it was experimentally challenging to fabricate them with the same low density, which led to similar thermal conductivity for both aerogel types (Table S3, Supporting Information). A more detailed analysis of the thermal conductivity of the aerogels is discussed in Section S6 of the Supporting Information.

The aerogels were intended to be printed as deformable 3D structures to be integrated into stretchable devices. The combination of Li salt (plasticizer) and GOPS (crosslinker) enhanced the stretchability in PEDOT:PSS thick films (Figure S6a–c, Supporting Information) and was thus exploited to endow thick filaments of printed aerogels with mechanical bendability (Figure 3c–e and Movie S4, Supporting Information). Since the soft aerogel pillars always break in or near the mechanical/adhesive clamps during tensile testing, the tensile failure strain of the aerogel pillars was determined by the bending strain (details provided in Material and Methods, Supporting Information). Considering that formulations *A1* and *A2* had the highest strain at failure in thick films (Figure S6a, Supporting Information), it is not surprising that we found their aerogel versions also to have the highest bending strain (12.1% and 24.1%, respectively). However, it should be noted that, unlike dense films, the aerogel version of *A2* is more stretchable than *A1*. This difference could be attributed to the influence of microstructure in the bending strain. Indeed, aerogel *A2* presented larger pores and lower density (more %vol of air) than aerogel *A1*, which presumably allowed superior accommodation of strain and led to enhanced filament bendability (Figure S3 and Table S3, Supporting Information). Conveniently, the filtration process not only improved the power factor of the pristine PEDOT:PSS but also its flexibility (increasing its maximum bending strain from 6.5% to 8.4%), presumably due to the removal of loose PSS, leading to an increased physical interaction between PEDOT fibers. We conducted cyclic mechanical bending tests and measured four‐point resistance to monitor changes in electrical resistance and assess the materials' endurance to cyclic mechanical stress (Figure 3e). All samples were bent around a 7.5 mm curvature radius, corresponding to an 8.4% bending strain. As expected, the reference aerogel *R* could not withstand a single cycle, while the filtered aerogel endured 70 cycles. Aerogels *A2* and *A1*, capable of withstanding the highest strains, performed best, surviving ≈450 and ≈200 cycles, respectively. Overall, the resistance changes were minimal across all samples during the cyclic tests, demonstrating the mechanical stability of the aerogels.

Through meticulous processing, including incorporating additives and filtration techniques, we have enhanced both the *ZT* and the maximum bending strain of DIW aerogels, as illustrated in Figure 3c. Our methodology facilitates the creation of a diverse library of DIW aerogels, each exhibiting a distinct balance between thermoelectric and mechanical properties. This material library enables application‐specific selection based on these properties. For instance, the filtration process notably increased the *ZT* but displayed only a moderate improvement in the maximum bending strain, making aerogel *F* particularly suitable for thermoelectric applications. Similarly, aerogel *A2* exhibits the highest bending strain but relatively lower ZT than F and is ideal for stretchable interconnects. Aerogel *A1*, offering a balanced tradeoff between *ZT* and bending strain, is well‐suited for applications requiring both stretchability and thermoelectric performance.

To understand how the utilized additives boost electrical transport, we examine the morphology of the different printed aerogels using synchrotron wide‐angle X‐ray scattering (WAXS) and Raman spectroscopy. 2D WAXS patterns are depicted in Figure 4a and their 1D line‐cut intensity profiles are presented in Figure 4b. The notable peaks observed around 1.2 and 1.8 Å⁻¹ across all samples are attributed to the PSS halo and the PEDOT π–π stacking distance (010), respectively.^[^ ^51^ ^]^ All our processed aerogels showed a shift of the (010) peak to *q* values higher than for the pristine PEDOT:PSS aerogel (*R*) used as a reference, suggesting a decrease in the π–π stacking distance ranging from 0.01 to 0.08 Å (Table S4, Supporting Information), consistent with what was reported for PEDOT:PSS treated with additives to improve conductivity.^[^ ^19^, ^21^, ^61^ ^]^ There is a direct link between increased electrical conductivity and the denser crystal packing resulting from a shorter π–π stacking distance. Closer molecules promote overlapping of the π orbital between neighboring molecules, facilitating smoother interchain transport and increasing electrical conductivity.^[^ ^61^, ^62^ ^]^ This correlation was interestingly linear and was confirmed for all our formulations, which displayed higher conductivities than the pristine material (Figure S10a, Supporting Information). In contrast, only a weak positive correlation was observed between the π–π stacking distance and the Seebeck coefficient (Figure S10b, Supporting Information). The aerogel *F*, from which the excess PSS was removed, displayed a comparatively large (010) peak. This can be attributed to the fact that the intensity of all the curves was normalized to the PSS peak (at ≈1.2 Å⁻¹). Furthermore, this sample also displayed the strongest (010) shift (0.08 Å), accompanied by an intense (100) type I peak attributed to the PEDOT:PSS lamellar structure.^[^ ^61^ ^]^ Both findings evidenced the strong beneficial effect in crystal ordering associated with the removal of excess PSS, which underpins the boost in conductivity observed for *F*. On the other hand, for those samples with additives (*A1* and *A2*), the emergence of a new lamella peak, the (100) type II (at ≈0.88 Å⁻¹), was observed. The type (100) type II peak has been previously reported for PEDOT:PSS doped with ionic liquids and was ascribed to a different PEDOT:PSS lamellar stacking in which adjacent PEDOT chains are staggered and closer to each other.^[^ ^21^, ^61^ ^]^ To verify that the origin of this new peak arises from the interaction between PEDOT and Li salt rather than from potential Li salt aggregates, we examined PSS films blended with Li salt with grazing incidence WAXS. No diffraction was detected at 0.88 Å⁻¹ for the bare PSS + Li salt film (Figure S10c, Supporting Information). Compared with type I, type II layer packing has been thought to facilitate charge transport between adjacent PEDOT chains, which results in improved electron conductivity.^[^ ^61^ ^]^ According to this analysis, the reduction in π–π stacking distance and the appearance of the type II lamella stacking can explain the enhanced electrical conductivity upon Li salt addition. Moreover, the increase in the PEDOT phase packing observed upon the addition of Li salt suggests that the Li salt molecules primarily occupy less ordered/amorphous regions, thereby softening them. This results in a morphology consisting of a crystalline nanofiber network surrounded by a soft matrix, which enhances the stretchability of the structure.^[^ ^21^ ^]^

The Raman spectra in Figure 4c reflect the PEDOT chain's conformation. The primary Raman peak, occurring between 1400 and 1500 cm^−1^, corresponds to the stretching vibration of *C_α_* = *C_β_* on the five‐member PEDOT ring.^[^ ^63^ ^]^ The presence of additives in our aerogels resulted in a redshift of this Raman peak.^[^ ^21^, ^61^ ^]^ Specifically, the peak position for aerogel *R* was at 1430 cm^−1^, while for aerogels *F*, *A1*, and *A2*, it was at 1428, 1426, and 1426 cm^−1^, respectively. This redshift trend matched the reported literature on PEDOT:PSS doped with secondary dopants or ionic species.^[^ ^21^, ^61^, ^63^ ^]^ In contrast, GOPS alone did not affect the Raman peak shift.^[^ ^64^ ^]^ Hence, we can conclude that the Li salt is the main responsible for the redshift of the peak signal, suggesting a conformational change in PEDOT from a coiled backbone conformation to a more extended backbone conformation. This occurs presumably due to the Li salt contributing to partially screening the strong coulombic interaction between PEDOT and coiled PSS^[^ ^19^, ^21^ ^]^ (Figure 4d). An extended backbone enhanced the planarity of PEDOT and facilitated the stacking of PEDOT chains, thereby promoting charge delocalization and efficient charge transport. This result confirmed the conclusions retrieved from the WAXS characterization.

### 2.3 Demonstrators

Screening the aerogel compositions in terms of thermoelectric and mechanical properties provided us with a material library to consult when a material is sought for specific applications. This library, combined with the shape freedom enabled by 3D printing, results in a powerful toolbox for innovative applications involving 3D soft electronics. To illustrate this concept, we developed three demos where the material properties matched a particular application: From the three processed aerogels, *A2* presents the best bendability but the lowest *ZT*. Hence, aerogel *A2* would be the most suitable material for applications where mechanical deformation prevails over TE performance, such as in stretchable interconnects (demo #1). Aerogel *A1* offers the best balance between TE performance and bendability, thereby fitting best the requirements of stretchable thermoelectrics (demo #2). Finally, aerogel *F* stands out for its superior *ZT* at the expense of modest mechanical stretchability. This makes *F* the best option for TE applications that do not require much mechanical strain (demo #3).

**Figure 5:**
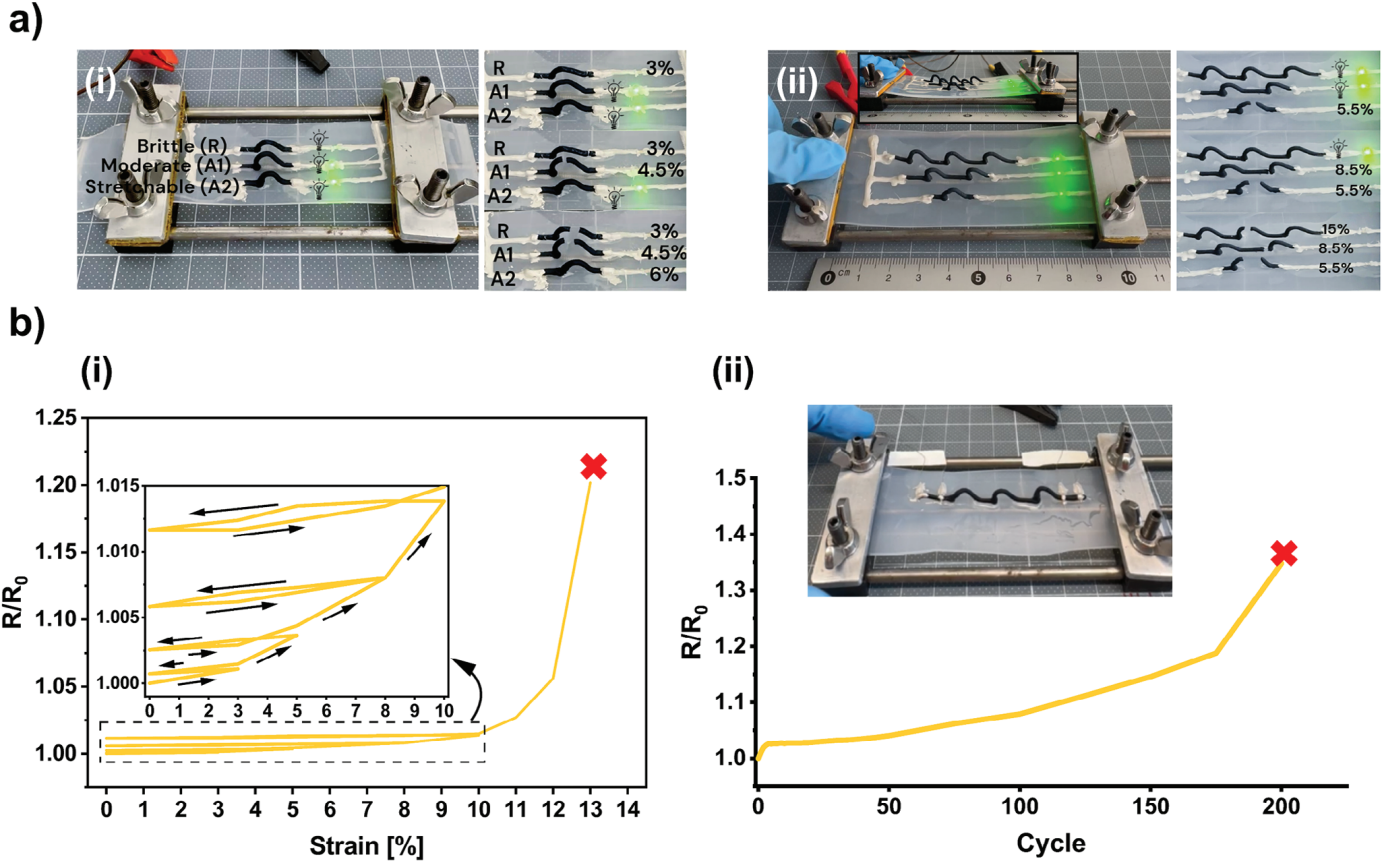
Demo #1: Stretchable interconnects based on through‐plane arches of aerogel 3D‐printed on an elastomer substrate. a) Stretchability test for i) different material compositions and ii) different device designs. b,i) The resistance change versus iterative applied strain and ii) cyclic strain and optical photograph of the stretching setup used with a demo consisting of a triple arch single line (aerogel *A2*) mounted in it.

**Figure** **5**ai depicts the experiment designed to confirm that *A2* is the most suitable aerogel for demo #1. We produced stretchable lines from our bendable aerogels by printing them as out‐of‐plane arches directly on an elastomeric substrate. The material composition could tune the device's stretchability. To prove this point, circuits composed of a single aerogel arch and stretchable Ag paste connecting to a small LED (Light‐emitting Diode) light were printed on a stretchable substrate. Upon strain application, the circuit containing the reference aerogel (*R*) failed at 3% strain (the LED switched off), while the circuits made with aerogels *A1* and *A2* withstood 4.5% and 6% strain, respectively (Movie S5, Supporting Information). Besides the material composition, the 3D shaping freedom offered by DIW could be exploited to tune stretchability. To illustrate this concept, we chose the most flexible material from our library (*A2*). Note that the relatively low thermoelectric performance of *A2* is not a bottleneck for this application. The aerogel was printed in three different circuits, each with a different number of arches (arch height ≈5 mm and length ≈7 mm) (Figure 5aii). The 3D printer G‐code is attached in the Supporting Information. In principle, the higher the number of arches, the larger the strain at circuit failure because the arches' deformation helps accommodate strain. As expected, the single‐arched line failed at 5.5% strain, followed by the double‐ and triple‐arched lines that failed at 8.5% and 15% strain, respectively (see Movie S6, Supporting Information). Although an optimized design maximizing strain was not the focus of this work, we showed that design can help enhance stretchability. In our proof of concept device consisting of 3D printed arches, the max strain increased as 5%/arch. The mechanical stability of the triple‐arched line was tested via iterative stretching to increasing strains (Figure 5bi and Movie S7, Supporting Information). This test shows the device's resistance change is very low, below 1.5%, up to the onset of failure ≈10% strain. After stretching beyond the previous maximum strain value, a mild hysteresis was observed in the contraction phase. The reason for this behavior is likely irreversible plastic deformation of the material upon stretching. To support the claim of the stretchability and stability of the 3D printed arches in the device configuration, devices have been exposed to cyclic 10% strain (≈75% of the maximum strain it can withstand). It has been observed that the device's resistance change under cyclic strain is very low, below 1.5%, up to the onset of failure after ≈200 cycles (Figure 5bi). It is important to note that the width and height of the aerogel arches can be adjusted and optimized based on the specific application. However, the primary goal of this study is not to demonstrate the most optimal configuration but rather to showcase the potential to tune both the mechanical and thermoelectric performances through changes in design and material composition.

**Figure 6:**
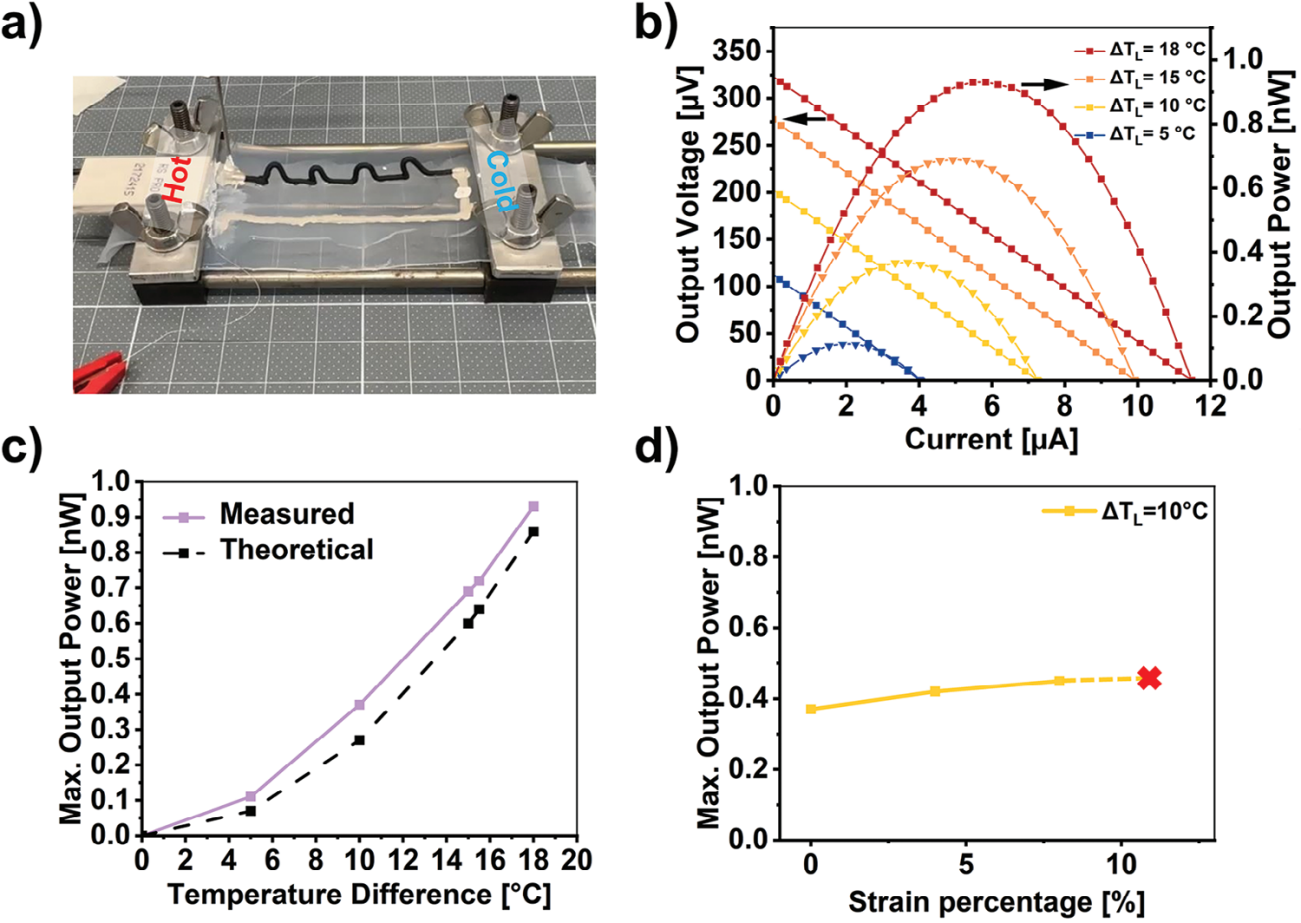
Demo #2: Stretchable planar thermoelectric generators based on 3D‐printed through‐plane arches. a) Optical photograph of a planar stretchable thermocouple composed of out‐of‐plane PEDOT:PSS (material composition *A1*) arches and painted stretchable silver. The device is mounted in a house‐made stretching set‐up and coupled to a Peltier unit at one end to generate a thermal gradient. b) Output voltage and output power versus output current for different resistive loads and temperature differences across the device. c) Maximum output power at different temperature differences across the device. d) Maximum output power versus applied strain for a 10 °C degree temperature difference across the device.

The 3D‐printed stretchable lines shown above can be repurposed as the p‐type leg of a stretchable thermocouple able to harvest waste heat, responding in this way to the standing challenge of stretchable electronics of finding mechanically complying power sources. A stretchable thermocouple could also work as a wearable self‐powered thermometer. For this second demo (demo #2), we selected aerogel *A1* for its balance between mechanical properties and thermoelectric performance. We completed the thermocouple with stretchable silver acting as the n‐type leg (although it is actually a poor p‐type material with a Seebeck coefficient of *S_n_ *= +6.5 µV K^−1^) (see **Figure** **6**a and Movie S8, Supporting Information). As depicted in Figure 6b, the thermocouple showed a typical thermoelectric generator behavior with the output power increasing quadratically with the temperature difference across the TE legs, Δ*T*_L_, (Figure 6c) according to the equation^[^ ^40^ ^]^

(1) $$P_{\max}=\frac{{\left[\left(S_{p}-S_{n}\right)\Delta T_{L}\right]}^{2}}{4R_{i}}\;$$

where *S*_p_ and *S*_n_ are the Seebeck coefficient of the PEDOT:PSS aerogel *A1* and the stretchable silver, respectively, and *R*_i_ is the internal resistance of the thermocouple (detailed explanation in Section S5, Supporting Information). A maximum output power of ≈0.95 nW was measured for a temperature difference of 18 °C. The thermocouple, subjected to a temperature difference of 10 °C, could be stretched up to 13% strain without performance loss (see Figure 6d). This strain invariability can also be understood theoretically (see Equation (S11)–(S14), Supporting Information, and corresponding analysis). The versatility of DIW permitted the change of the PEDOT: PSS's layout freely. To illustrate this, another demo with a similar performance was developed. The PEDOT:PSS leg was shaped as a planar serpentine structure instead of as out‐of‐plane arches (see Figure S9 and Movie S9, Supporting Information).

Aerogels could be suitable for thermoelectric applications because their low thermal conductivity, resulting from their porous structure, can enhance the figure of merit *ZT*. However, this same porous structure lowers the electrical conductivity even further than the thermal conductivity (21.5‐fold vs 8.5‐fold for our materials), resulting in a *ZT* for aerogels almost 2.5 times lower than their denser counterparts. While this situation might discourage the use of aerogel for thermoelectrics, in real‐world applications, the electrical and thermal transport is heavily influenced by unavoidable contact resistances, which can alter the relative importance of *ZT*. Contact resistances strongly harm the performance of TE generators and should be minimized, but they are challenging to eliminate in small devices like the one proposed in this study. A detailed analysis of the maximum output power provided by a TE leg, *P*_max_, with electrical and thermal contact resistances, *R*_C_ and (*R’*_H_ + *R’*_C_) respectively, taken into consideration, leads to the following variation of Equation (1) (see Equations (S15)–(S21), Figure S12, and corresponding analysis, Supporting Information)

(2)

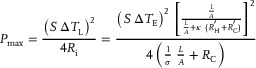

where Δ*T*_E_ is the temperature difference applied externally to the device and Δ*T*_L_ is the actual temperature difference across the TE leg; *R*_i_ is the internal electrical resistance of the device, composed of the net contribution from the TE leg and the electrical contact resistance *R*_C_; *R’*_H_ and *R’*_C_ are the thermal contact resistance for hot and cold end, respectively; *L* and *A* are the length and cross‐sectional area of the TE leg; *S* is the Seebeck coefficient; and *σ* and *κ* are the electrical and thermal conductivity of the material, respectively. According to Equation (2), in devices presenting high electrical and thermal contact resistance, a material with high *σ* and *κ* would produce less power than a counterpart with low *σ* and *κ*, even if the former has a comparatively higher *ZT*. We illustrate this situation in Figure S13 in the Supporting Information for different materials, including those comparable to our dense 0* and aerogel *F* formulations, subjected to idealistic and realistic *R*_C_ and (*R’*_C_+ *R’*_H_) scenarios. The reason for this counterintuitive behavior is twofold: On one hand, the benefit of high *σ* is masked by a dominant electrical contact resistance; on the other hand, a low *κ* leads to a more significant temperature difference across the TE leg, Δ*T*_L_, for the same externally applied temperature difference, Δ*T*_E_ *¸* (Figure S14, Supporting Information) and the larger the thermal contact resistance, the more marked this behavior is. The latter aspect was already highlighted in the seminal paper by Suarez et al. within the context of skin‐mounted TEs, in which a large thermal contact resistance exists between the human skin and the hot end of the TE generator and between the cold end of the TE generator and the surrounding air.^[^ ^65^ ^]^ Furthermore, the benefit of a lower *κ* is magnified in wearables and other applications that cannot afford to use bulky heat sinks on the cold side to reduce the thermal contact resistance with the air.

**Figure 7:**
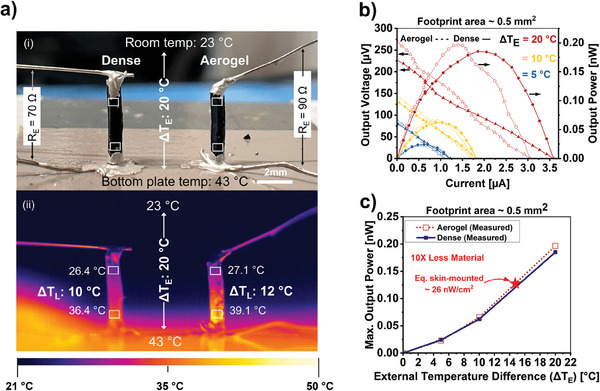
Demo #3: Thermoelectric performance of 3D printed pillars. Comparison between dense material and aerogel. a) Optical image of the two pillars side‐by‐side showing the electrical connections. i) The applied external temperature across the device (Δ*T*_E_), as well as the measured total internal electrical resistance of the device, are indicated and ii) a representative frame obtained with a thermal cameral displaying the actual temperature difference across the TE leg (Δ*T*_L_). b) Output voltage and output power change versus output current for different resistive loads and temperature differences across the device. c) Maximum output power comparison at different external temperature differences across the device.

The previous analysis suggests the promise of using aerogels in TE generators. To confirm this point experimentally, we printed a pillar made of the dense 0* PEDOT:PSS next to another pillar made of the aerogel *F*, both with the same dimensions, *L* = 3.1 mm (distance between electrodes) and *A* = 0.5 mm^2^ (cross‐sectional area), (demo #3 depicted in **Figure** **7**ai). To reach the same diameter for both dry pillars, the 0* pillar was originally printed with a larger diameter to account for its shrinkage upon drying. The formulation *F* was used in demo #3 because it is the material from our library with the best *ZT* and its poor mechanical performance is not an issue in this scenario. The pillars were welded to a thermally conducting soft substrate using Ag paste to mimic a realistic skin electronics scenario. They were electrically interconnected at the top and bottom using the same Ag paste. The measured internal resistances for dense and aerogel interconnected pillars were 70 and 90 Ω, respectively. Since the calculated net material resistance (1/*σ L/A*) for the aerogel and the dense legs is only 4.8 and 0.2 Ω, respectively, the contact resistance dominated the internal resistance. The demo #3 was placed on a hotplate to heat the bottom end of the pillars while the top was left free to cool down by normal air convection/conduction. The external temperature (Δ*T*_E_) was measured using a thermometer and a thermocouple, and the temperature difference across the leg (Δ*T*_L_) was monitored with a thermal camera, as illustrated in Figure 7aii. It could be appreciated in the thermal images that, as predicted by Equation (2), Δ*T*_L_ was more significant for the aerogel than for the dense pillar, even though both pillars were subjected to the same Δ*T*_E_. The curves in Figure 7b display the typical behavior of TE generators for both pillars, where the output voltage and output power increased linearly and quadratically (Figure 7c), respectively, with Δ*T*_E_. Interestingly, despite the aerogel's *ZT* being less than half the *ZT* of the dense material (2.4 × 10^−3^ vs 6.0 × 10^−3^), the aerogel pillar could produce more output power than the dense pillar because it could hold a more significant temperature gradient across itself.

An areal power density of 26 nW cm^−2^ was achieved for the aerogel at Δ*T*_E_ = 15 °C, mimicking the conditions of indoor skin electronics (substrate at 37 °C and air at 22 °C). This power density value is comparable to the best‐reported value for organic TEs in the existing literature (Table S6, Supporting Information)^[^ ^39^, ^66^ ^]^ despite requiring ten times less material. When computing the gravimetric power density, a merit that we expect to become increasingly relevant for wearables and small autonomous systems, the aerogel reached a value of 7.6 × 10^−4^ W kg^−1^ (Ag paste is ignored in the calculation).

Our results support the potential of aerogels for out‐of‐plane thermoelectric devices, especially for wearables and many other cases requiring low power and in which miniaturization makes it challenging to reduce electrical contact resistance, the thermal interface between the device and the hot surface cannot be optimized, or the lack of a heat sink at the cold side leads to a high thermal contact resistance at the cold side.

## 3 Conclusions

This study showed the possibility of omnidirectional 3D printing of PEDOT:PSS aerogels with tunable mechanical and electrical performance achieved via different paste formulations. We developed a process flow that allowed us to direct ink write hydrogels on top of a stretchable substrate with sufficient attachment to survive a subsequent freeze‐dying step. The freeze‐drying turned the hydrogel into an aerogel, which preserved enough adhesion to the substrate to survive joint straining. In this way, the resulting aerogels integrated into the stretchable substrate demonstrated unconventional stretchable interconnects and thermoelectric devices. The devices' mechanical and thermoelectric performance could be tuned to adapt to different applications by 1) material composition/formulation and 2) the freedom of 3D printing design. We illustrated this versatility through three demonstrations: stretchable interconnects (demo #1), a stretchable planar thermocouple (demo #2), and a vertical thermoelectric generator (demo #3). A comparison between the thermoelectric performance of a dense and an aerogel pillar revealed that utilizing aerogels is advantageous for energy harvesting in the presence of contact resistances.

While our current 3D‐printed PEDOT:PSS aerogels present significant potential for soft electronics and robots (stretchable 3D interconnects, skin‐mounted self‐powered thermometers, wearable energy harvesters, etc.), further research is needed to enhance their mechanical robustness and overall performance for practical use. Potential future improvements include encapsulating the devices with stretchable elastomers to improve durability, printing parameters, and formulation optimization,^[^ ^67^ ^]^ fine‐tuning the design of the printed structure to minimize stress concentration points, and incorporating carbon nanotubes (CNTs)^[^ ^68^ ^]^ or optimizing post‐treatment processes^[^ ^17^ ^]^ to boost both mechanical and thermoelectric performance. These strategies will be crucial in advancing the practical viability of these materials.

## Conflict of Interest

The authors declare no conflict of interest.

## Author Contributions

Author contributions are defined based on the CRediT (Contributor Roles Taxonomy) and listed alphabetically: conceptualization (F.M.‐L., H.E.B.); data curation (F.M.‐L., H.E.B.); formal analysis (F.M.‐L., H.E.B., T.‐Y.Y., V.N., S.D.S., B.Z., H.X., R.C.); funding acquisition (F.M.‐L., R.C.); investigation (F.M.‐L., H.E.B., T.‐Y.Y., V.N., S.D.S., D.H., B.Z., H.X.); methodology (F.M.‐L., H.E.B., T.‐Y.Y., V.N., S.D.S., B.Z., H.X., I.F., M.R., R.C.); project administration (F.M.‐L.); supervision (F.M.‐L., M.R., R.C.); validation (F.M.‐L., H.E.B., T.‐Y.Y., V.N., S.D.S., B.Z., H.X., M.R., R.C.); visualization (F.M.‐L., H.E.B.); writing—original draft (F.M.‐L., H.E.B.); writing—review and editing (F.M.‐L., H.E.B., T.‐Y.Y., V.N., S.D.S., D.H., B.Z., H.X., I.F., M.R., R.C.).

## Supporting information



Supporting Information

Supplemental Movie 1

Supplemental Movie 2

Supplemental Movie 3

Supplemental Movie 4

Supplemental Movie 5

Supplemental Movie 6

Supplemental Movie 7

Supplemental Movie 8

Supplemental Movie 9

Supplemental Movie 10

## Data Availability

The data that support the findings of this study are available from the corresponding author upon reasonable request.

## References

[1] C. Wang , C. Wang , Z. Huang , S. Xu , Adv. Mater. 2018, 30, 1801368. 10.1002/adma.201801368 30073715

[2] S. I. Rich , R. J. Wood , C. , Majidi , Nat. Electron. 2018, 1, 102.

[3] M. Lin , H. Hu , S. Zhou , S. Xu , Nat. Rev. Mater. 2022, 7, 850.

[4] H. R. Lim , H. S. Kim , R. Qazi , Y. T. Kwon , J. W. Jeong , W. H. Yeo , Adv. Mater. 2020, 32, 1901924. 10.1002/adma.201901924 31282063

[5] M. H. Kim , C. H. Cho , J. S. Kim , T. U. Nam , W. S. Kim , T. Il Lee , J. Y. Oh , Nano Energy 2021, 87, 106156.

[6] C. García Núñez , L. Manjakkal , R. Dahiya , npj Flexible Electron. 2019, 3, 1.

[7] S. Zhang , S. Li , Z. Xia , K. Cai , J. Mater. Chem. B 2020, 8, 852. 31942905 10.1039/c9tb02531f

[8] T. Distler , A. R. Boccaccini , Acta Biomater. 2020, 101, 1. 31476385 10.1016/j.actbio.2019.08.044

[9] Y. Tian , F. Molina‐Lopez , Nanoscale 2021, 13, 5202. 33688886 10.1039/d0nr08144b

[10] W. Liu , H. S. Kim , Q. Jie , Z. Ren , Scr. Mater. 2016, 111, 3.

[11] I. T. Witting , T. C. Chasapis , F. Ricci , M. Peters , N. A. Heinz , G. Hautier , G. J. Snyder , Adv. Electron. Mater. 2019, 5, 1800904.

[12] M. M. Mallick , L. Franke , A. G. Rösch , H. Geßwein , Z. Long , Y. M. Eggeler , U. Lemmer , Adv. Sci. 2022, 9, 2202411. 10.1002/advs.202202411 PMC9631075 36106362

[13] H. Cho , D. Jang , J. Yoon , Y.‐S. Ryu , B. Lee , B. Lee , S. Chung , Y. Hong , ACS Energy Lett. 2023, 8, 2585.

[14] F. Molina‐Lopez , in Proceedings of IEEE Sensors , Vol. 2020‐Octob, IEEE, Rotterdam 2020, pp 1–4.

[15] Y. Tian , I. Florenciano , H. Xia , Q. Li , H. E. Baysal , D. Zhu , E. Ramunni , S. Meyers , T. Y. Yu , K. Baert , T. Hauffman , S. Nider , B. Göksel , F. Molina‐Lopez , Adv. Mater. 2024, 36, 2307945. 10.1002/adma.202307945 38100238

[16] D. Zhang , W. Y. S. Lim , S. S. F. Duran , X. J. Loh , A. Suwardi , ACS Energy Lett. 2022, 7, 720.

[17] Z. Fan , J. Ouyang , Adv. Electron. Mater. 2019, 5, 1800769.

[18] G. H. Kim , L. Shao , K. Zhang , K. P. Pipe , Nat. Mater. 2013, 12, 719. 23644522 10.1038/nmat3635

[19] D. Ju , D. Kim , H. Yook , J. W. Han , K. Cho , Adv. Funct. Mater. 2019, 29, 1905590.

[20] U. Kraft , F. Molina‐Lopez , D. Son , Z. Bao , B. Murmann , Adv. Electron. Mater. 2020, 6, 1900681.

[21] Y. Wang , C. Zhu , R. Pfattner , H. Yan , L. Jin , S. Chen , F. Molina‐Lopez , F. Lissel , J. Liu , N. I. Rabiah , Z. Chen , J. W. Chung , C. Linder , M. F. Toney , B. Murmann , Z. Bao , Sci. Adv. 2017, 3, e1602076. 28345040 10.1126/sciadv.1602076 PMC5345924

[22] X. Fan , W. Nie , H. Tsai , N. Wang , H. Huang , Y. Cheng , R. Wen , L. Ma , F. Yan , Y. Xia , Adv. Sci. 2019, 6, 1900813. 10.1002/advs.201900813 PMC6774040 31592415

[23] O. Jin Young , S. Kim , H. K. Baik , U. Jeong , Adv. Mater. 2016, 28, 4455. 26460551 10.1002/adma.201502947

[24] A. Håkansson , S. Han , S. Wang , J. Lu , S. Braun , M. Fahlman , M. Berggren , X. Crispin , S. Fabiano , J. Polym. Sci., Part B: Polym. Phys. 2017, 55, 814.

[25] Z. U. Khan , J. Edberg , M. M. Hamedi , R. Gabrielsson , H. Granberg , L. Wågberg , I. Engquist , M. Berggren , X. Crispin , Adv. Mater. 2016, 28, 4556. 26836440 10.1002/adma.201505364

[26] Q. Wei , M. Mukaida , K. Kirihara , T. Ishida , ACS Macro Lett. 2014, 3, 948. 35596366 10.1021/mz500446z

[27] J. Liu , X. Wang , D. Li , N. E. Coates , R. A. Segalman , D. G. Cahill , Macromolecules 2015, 48, 585.

[28] S. Xu , M. Li , M. Hong , L. Yang , Q. Sun , S. Sun , W. Lyu , M. Dargusch , J. Zou , Z. G. Chen , J. Mater. Sci. Technol. 2022, 124, 252.

[29] P. Sun , J. Zhang , X. Zhu , H. Li , Y. Li , J. Yang , Z. Peng , G. Zhang , F. Wang , H. Lan , Adv. Mater. Technol. 2022, 7, 2200302.

[30] J. Li , J. Cao , B. Lu , G. Gu , Nat. Rev. Mater. 2023, 8, 604.

[31] W. Monnens , B. Zhang , Z. Zhou , L. Snels , K. Binnemans , F. Molina‐Lopez , J. Fransaer , Adv. Mater. 2023, 35, 2305967. 10.1002/adma.202305967 37703420

[32] A. D. Valentine , T. A. Busbee , J. W. Boley , J. R. Raney , A. Chortos , A. Kotikian , J. D. Berrigan , M. F. Durstock , J. A. Lewis , Adv. Mater. 2017, 29, 1703817. 10.1002/adma.201703817 28875572

[33] B. Lee , H. Cho , S. Moon , Y. Ko , Y. S. Ryu , H. Kim , J. Jeong , S. Chung , Nat. Electron. 2023, 6, 307.

[34] R. S. Jordan , Y. Wang , J. Polym. Sci., Part B: Polym. Phys. 2019, 57, 1592.

[35] J. Liu , L. Mckeon , J. Garcia , S. Pinilla , S. Barwich , M. Möbius , P. Stamenov , J. N. Coleman , V. Nicolosi , Adv. Mater. 2022, 34, 2106253. 10.1002/adma.202106253 34784072

[36] W. Xing , J. Wang , Q. Qian , C. Wang , H. Guo , W. Tan , J. Wu , H. Tang , H. Qi , H. Lin , ACS Appl. Mater. Interfaces 2023, 15, 57717. 10.1021/acsami.3c14851 38018535

[37] F. Kim , S. E. Yang , H. Ju , S. Choo , J. Lee , G. Kim , S. Jung , S. Kim , C. Cha , K. T. Kim , S. Ahn , H. G. Chae , J. S Son , Nat. Electron. 2021, 4, 579.

[38] C. K. Mytafides , W. J. Wright , R. Gustinvil , L. Tzounis , G. Karalis , A. S. Paipetis , E. Celik , Energy Adv. 2024, 3, 1642.

[39] M. Massetti , S. Bonfadini , D. Nava , M. Butti , L. Criante , G. Lanzani , L. Qiu , J. C. Hummelen , J. Liu , L. J. A. Koster , M. Caironi , Nano Energy 2020, 75, 104983.

[40] M. Mallick , L. Franke , A. G. Rösch , U. Lemmer , ACS Energy Lett. 2021, 6, 85.

[41] P. A. Amorim , M. A. d’Ávila , R. Anand , P. Moldenaers , P. Van Puyvelde , V. Bloemen , Bioprinting 2021, 22, e00129.

[42] A. Chortos , J. Polym. Sci. 2022, 60, 486.

[43] H. Yuk , B. Lu , S. Lin , K. Qu , J. Xu , J. Luo , X. Zhao , Nat. Commun. 2020, 11, 4. 32231216 10.1038/s41467-020-15316-7 PMC7105462

[44] T. Zhou , H. Yuk , F. Hu , J. Wu , F. Tian , H. Roh , Z. Shen , G. Gu , J. Xu , B. Lu , X. Zhao , Nat. Mater. 2023, 22, 895. 37322141 10.1038/s41563-023-01569-2

[45] T. Li , Y. Huang , J. X. M. Chen , Y. C. Sun , O. Aghababaei , Z. Saadatnia , H. E. Naguib , Nano Energy 2023, 117, 108909.

[46] Q. Weinbach , S. V. Thakkar , A. Carvalho , G. Chaplais , J. Combet , D. Constantin , N. Stein , D. Collin , L. Biniek , Front. Electron. Mater. 2022, 2, 875856.

[47] L. Chen , J. Lou , Y. Zong , Z. Liu , Y. Jiang , W. Han , Cellulose 2023, 30, 3141.

[48] G. Chen , R. Rastak , Y. Wang , H. Yan , V. Feig , Y. Liu , Y. Jiang , S. Chen , F. Lian , F. Molina‐Lopez , L. Jin , K. Cui , J. W. Chung , E. Pop , C. Linder , Z. Bao , Matter 2019, 1, 205.

[49] M. P. Gordon , E. W. Zaia , P. Zhou , B. Russ , N. E. Coates , A. Sahu , J. J. Urban , J. Appl. Polym. Sci. 2017, 134, 44070.

[50] J. Nevrela , M. Micjan , M. Novota , S. Kovacova , M. Pavuk , P. Juhasz , J. Kovac , J. Jakabovic , M. Weis , J. Polym. Sci., Part B: Polym. Phys. 2015, 53, 1139.

[51] A. C. Hinckley , S. C. Andrews , M. T. Dunham , A. Sood , M. T. Barako , S. Schneider , M. F. Toney , K. E. Goodson , Z. Bao , Adv. Electron. Mater. 2021, 7, 2001190.

[52] H. Wang , T. Zhuang , J. Wang , X. Sun , Y. Wang , K. Li , X. Dai , Q. Guo , X. Li , D. Chong , B. Chen , J. Yan , Adv. Mater. 2023, 35, 2302919. 10.1002/adma.202302919 37352335

[53] S. Zhang , Y. Chen , H. Liu , Z. Wang , H. Ling , C. Wang , J. Ni , B. Çelebi‐Saltik , X. Wang , X. Meng , H. J. Kim , A. Baidya , S. Ahadian , N. Ashammakhi , M. R. Dokmeci , J. Travas‐Sejdic , A. Khademhosseini , Adv. Mater. 2020, 32, 1904752. 10.1002/adma.201904752 PMC6946856 31657081

[54] H. E. Baysal , F. Molina‐Lopez , in Proc. 2023 IEEE Int. Flex. Electron. Technol. Conf. (IFETC) , San Jose, CA, USA, 2023, pp. 1–3.

[55] S. Han , F. Jiao , Z. U. Khan , J. Edberg , S. Fabiano , X. Crispin , Adv. Funct. Mater. 2017, 27, 1703549.

[56] T. A. Yemata , Y. Zheng , A. K. K. Kyaw , X. Wang , J. Song , W. S. Chin , J. Xu , Mater. Adv. 2020, 1, 3233. 10.1039/c9ra07648d PMC9047250 35494687

[57] T. Ji , L. Tan , X. Hu , Y. Dai , Y. Chen , Phys. Chem. Chem. Phys. 2015, 17, 4137. 25563771 10.1039/c4cp04965a

[58] A. Bisson , A. Rigacci , D. Lecomte , E. Rodier , P. Achard , Drying Technol. 2003, 21, 593.

[59] J. Park , J. G. Jang , K. Kang , S. H. Kim , J. Kwak , Adv. Sci. 2024, 11, 2308368. 10.1002/advs.202308368 PMC10933597 38236169

[60] O. Bubnova , Z. U. Khan , H. Wang , S. Braun , D. R. Evans , M. Fabretto , P. Hojati‐Talemi , D. Dagnelund , J. B. Arlin , Y. H. Geerts , S. Desbief , D. W. Breiby , J. W. Andreasen , R. Lazzaroni , W. M. Chen , I. Zozoulenko , M. Fahlman , P. J. Murphy , M. Berggren , X. Crispin , Nat. Mater. 2014, 13, 190. 24317188 10.1038/nmat3824

[61] X. Li , R. Zou , Z. Liu , J. Mata , B. Storer , Y. Chen , W. Qi , Z. Zhou , P. Zhang , npj Flexible Electron. 2022, 6, 6.

[62] F. Molina‐Lopez , H. C. Wu , G. J. N. Wang , H. Yan , L. Shaw , J. Xu , M. F. Toney , Z. Bao , Adv. Electron. Mater. 2018, 4, 1800110.

[63] P. Zhang , N. Aydemir , M. Alkaisi , D. E. Williams , J. Travas‐Sejdic , ACS Appl. Mater. Interfaces 2018, 10, 11888. 29570263 10.1021/acsami.8b02289

[64] A. X. Chen , G. L. Esparza , I. Simon , S. P. Dunfield , Y. Qie , J. A. Bunch , R. Blau , A. Lim , H. Zhang , S. E. Brew , F. M. O'Neill , D. P. Fenning , D. J. Lipomi , ACS Appl. Mater. Interfaces 2023, 15, 38143. 37499172 10.1021/acsami.3c08341

[65] F. Suarez , A. Nozariasbmarz , D. Vashaee , M. C. Öztürk , Energy Environ. Sci. 2016, 9, 2099.

[66] O. Bubnova , Z. U. Khan , A. Malti , S. Braun , M. Fahlman , M. Berggren , X. Crispin , Nat. Mater. 2011, 10, 429. 21532583 10.1038/nmat3012

[67] K. Song , G. Xu , A. N. M. Tanvir , K. Wang , M. O. Bappy , H. Yang , W. Shang , L. Zhou , A. W. Dowling , T. Luo , Y. Zhang , J. Mater. Chem. A 2024, 12, 21243.

[68] X. He , J. Shi , Y. Hao , L. Wang , X. Qin , J. Yu , Compos. Commun. 2021, 27, 100822.

