# Supplementary material for: Omnidirectional 3D Printing of PEDOT: PSS Aerogels with Tunable Electromechanical Performance: A Playground for Unconventional Stretchable Interconnects and Thermoelectrics
Source: Adv Sci (Weinh). 2025 Jan 22;12(11):2412491. doi: 10.1002/advs.202412491 (PMC11923886; doi:10.1002/advs.202412491)
Supplement: Supplementary file 1 — Supporting Information [file ADVS-12-2412491-s005.docx]

**Supplementary Information**

**OMNIDIRECTIONAL 3D PRINTING OF PEDOT:PSS AEROGELS WITH TUNABLE ELECTROMECHANICAL PERFORMANCE: A PLAYGROUND FOR UNCONVENTIONAL STRETCHABLE INTERCONNECTS AND THERMOELECTRICS**

Hasan Emre Baysal, Tzu-Yi Yu, Viktor Naenen, Stijn De Smedt, Defne Hiz, Bokai Zhang, Heyi Xia, Isidro Florenciano, Martin Rosenthal, Ruth Cardinaels, Francisco Molina-Lopez

**1. Materials and Methods:**

**Direct ink Writing (DIW) paste preparation.** For the DIW paste preparation, the method in our previous study^[1]^ was modified. For the non-filtered material, a commercially available Poly(3,4-ethylenedioxythiophene) polystyrene sulfonate (PEDOT:PSS) aqueous dispersion (Clevios^TM^ PH1000, Heraeus Electronic Materials) was mixed with the Li salt plasticizer bis(trifluoromethane)sulfonimide lithium salt (Alfa Aesar, CAS number: 90076-65-6) at the ratio indicated in Table S1 in an open Teflon container, and magnetically stirred overnight at room temperature. Later, the temperature was raised to 60°C, and the mixture was continuously stirred with a magnetic bar until reaching the target weight of 1.42 g (including Li salt) per 10 ml initial PEDOT:PSS dispersion. In the last step, the crosslinker (3-Glycidyloxypropyl)trimethoxysilane (GOPS) (Sigma Aldrich, CAS number: 2530-83-8) was added to the paste and stirred manually at room temperature before printing. GOPS was added at the end to prevent premature crosslinking impeaching DIW.

For the filtered samples, 15 ml of the PEDOT:PSS aqueous dispersion was diluted in 25 ml of dimethyl sulfoxide (DMSO) (Sigma-Aldrich, CAS number: 67-68-5) and stirred for 96 hours at 60°C in a closed container. The mixture was poured on top of a customized vacuum-assisted filtration and mixing system. The system continuously stirred the dispersion with a Teflon spatula to avoid film formation and aggregation on top of a 200 nm pore-size filter paper (NL16 Whatman Polyamide Membrane, Cytiva) while promoting the filtration of the extra solvent with dissolved excess PSS to be discarded (Movie S10). The dispersion was washed five times with 45 ml of deionized water, which was iteratively filtered out to remove the excess PSS (byproduct) and replace DMSO with water as the dispersion medium for the subsequent freeze-drying step.

**UV-visible spectroscopy.** To confirm the effective removal of PSS during filtration, the byproduct excess PSS solution and a commercial PEDOT:PSS dispersion was tested with a UV-visible spectrometer (UV1601, Shimadzu). The test liquids were drop-cast on quartz substrates and dried at room temperature to form test films.

**Rheology.** Rheological tests were conducted on an MCR501 stress-controlled rheometer (Anton Paar, Austria) with a Peltier temperature unit controlled at 25 °C. A roughened set of parallel plates with a diameter of 8 mm was used to avoid slip. The gap was set at 0.45 mm, and a mineral oil ring was placed around the sample to minimize water evaporation. The linear regime of each sample was determined in terms of deformation with an oscillatory amplitude sweep at 10 rad/s and deformations ranging from 0.1 % to 100 %. Dynamic frequency sweeps in the linear regime were performed at 1 % strain. Rotational rate sweeps were conducted to create flow curves. The shear rate was increased stepwise from 0.01 s^-1^ to 100 s^-1^ at 90 s per shear rate, resulting in steady state flow. Additionally, creep and recovery tests were performed to test the elastic recovery after applying a constant shear stress of 50 Pa for one hour.

**Test film preparation.** For thick film preparation to test thermoelectric parameters and mechanical properties, the PEDOT:PSS aqueous dispersion (with Li salt and/or GOPS, see Table S1) was magnetically stirred for an hour, the homogeneous dispersion was drop cast on a petri dish and degassed in a desiccator for half an hour before letting it dry at ambient conditions. The dry films (with thickness values ranging from 30-80 µm) were cut in test coupons, sandwiched between two metal plates (to keep them flat), and annealed on a hot plate at 120°C for an hour to promote the GOPS-mediated crosslinking. Later, the metal-sandwiched test coupons were post-treated via ethanol vapor annealing and heat, similar to the aerogels.

To prepare thin film reference samples, 1.5 cm x 1.5 cm glass slides were cleaned by sonicating sequentially with ethanol, isopropyl alcohol, and deionized water. Different compositions of PEDOT:PSS mixtures were spin-coated on the glass slides at 1000 rpm for 120 seconds and annealed on a hot plate at 120°C for an hour. Later, Pt/Pd electrodes were sputtered on top (Q150T S, Quorum). The film thickness was measured with a profilometer (Formtracer CS-3200, Mitutoyo).

**Mechanical testing.** The mechanical properties of these films were tested based on the ASTM norm D882. The samples were cut into 70 mm x 5 mm parts (with scissor/rotary trimmer-Dahle 552). The samples’ width and length were measured by an optical microscope (FHD Trend, Tagarno), and the thickness was measured by a digital micrometer. Universal tensile machine (UTM) tests were performed on an Instron 5943 system with air-actuated grips (Instron 2712-019): strain rate 0.05 %/s; 30-50 mm grips separation and gauge length. Samples with anomalous failure, particularly those breaking in or near the grip area, were excluded when determining the failure strain but not for determining Young’s modulus. Young’s modulus was defined as the maximum tangent modulus within the linear deformation region to minimize errors from the pronounced toe regions (i.e., the initial nonlinear regions) observed in the stress-strain curves of our samples. The toe region was corrected to extract the strain-zero stress point and, hence, get an accurate strain at the failure value according to the standard ASTMD882. Failure to follow this protocol might lead to underestimating Young’s modulus and overestimating the strain at failure.

To test the flexibility of the aerogels, the maximum bending strain was determined by bending 1.5 cm-long aerogel pillars with a diameter of 1.4 mm around cylinders with various radii. The maximum bending strain (tensile strain at the surface) was calculated as the ratio of pillar radius (half the thickness) to the actual bending radius at the neutral axis (radius of the cylinder plus radius of the pillar). The change in resistance was recorded with the two-wire technique while bending. For the cyclic tests on the aerogels, the same setup was utilized, and aerogel pillars were bent manually around the curvature while four-wire resistance was measured.

**Scanning Electron Microscopy (SEM).** The morphology of the samples was characterized by an FEI XL 30 FEG scanning electron microscope. For a fair comparison, all the samples were observed at 10 kV. The aerogel pore diameter was measured from SEM images using the image processing tool ImageJ.

**Atomic Force Microscopy (AFM).** The surface morphology of the samples was characterized by an AFM (NaioAFM, Nanosurf, Switzerland).

**Wide angle X-ray scattering (WAXS).** WAXS measurements were performed at the beamline BM26 (DUBBLE), European Synchrotron Radiation Facility (ESRF). Samples were probed by 12 keV X-ray with a 30-second exposure time. The sample-to-detector distance was 348.85 mm. Calibration was done with the α-Al_2_O_3_ standard. A helium fly-tube was placed between the sample and detector to reduce air scattering. 2D WAXS images were collected by a Pilatus 1M detector and visualized by GIuSAXS, a visualization tool funded by DUBBLE@ESRF. WAXS data were further reduced to 1D line-cut (integrated over an azimuthal angle χ = 180°) by “BUBBLE,” a data reduction software supported by DUBBLE@ESRF.

**Raman Spectroscopy.** The presented Raman spectra were collected using a Horiba LabRAM HR Evolution. Raman shift was calibrated with a NIST-certified Horiba SP-RCO calibration objective. The samples were probed with a 1.7 W, 633 nm wavelength HeNe laser with a 30-second exposure time. For each sample, the presented spectrum is the average of six spectra collected from three different spots. The spectra are fitted in Matlab by the nonlinear least-square solver *lsqcurvefit*. The pseudo-Voigt function was applied to identify peak position, intensity, and full-width-at-half-maximum (FWHM).

**Thermoelectric Characterization**

Conductivity and Seebeck coefficient, manual setup (room temperature): Electrical resistance measurements were done with the four-wires technique at room temperature with a multimeter (2000 Multimeter, KEITHLEY) connected to a probe station (SimplePS TRIAX, KeyFactor). Contact pads made of silver paste (LOCTITE^®^ ECI 1501 E&C, HENKEL) were deposited on the aerogel sample to improve the reliability of the electrical contact. To calculate the electrical conductivity of the printed aerogel pillars, their cross-sections, and distances between the contact pads were measured with an optical microscope (Prestige Digital Microscope, Tagarno). The Seebeck coefficient was calculated with a house-made Seebeck setup. The setup consists of two Peltier elements to apply a symmetric difference in temperature (around room temperature) across the sample, thermocouples to measure the temperature, and a multimeter to measure the thermo-voltage generated through the sample. The same leads were used to measure temperature and voltage to avoid the “cold finger” effect^[2]^. A correction factor of + 7 μV/K was applied to the measured sample’s Seebeck to account for the Seebeck coefficient effect of the leads. Contrary to the aerogel samples, no silver paste was applied to the films as it did not attach well to their surfaces.

Conductivity and Seebeck coefficient, automatic setup (temperature sweep)*:* The room temperature values of electrical conductivity and Seebeck coefficients measured with the manual setup were confirmed with a commercial setup LSR (LSR-3, Linseis). The measurements were also extended to a wide temperature range between -10 and 90 °C. These measurements took place under a 0.05 bar He atmosphere. Measuring in an inert atmosphere ensured the absence of potential local redox reactions at the metal contact/PEDOT:PSS interface that has been recently reported to influence the Seebeck measurements when the samples are measured in the presence of humidity^[3]^.

Thermal properties*:* Thermal diffusivity was measured under room temperature conditions and a nitrogen purge gas pressure of 0.5 bar with a light flash analyzer (LFA 467 Hyper Flash, Netzsch). The samples, prepared by blade coating the DIW paste followed by freeze drying, were mounted in a through-plane sample holder and coated locally with graphite spray to maximize heat absorption upon light exposure. The material’s specific heat (around room temperature) was measured by differential scanning calorimetry (DSC Q2000, TA Instruments) using hermetic crucibles to prevent moisture loss and match the real-application conditions in this way. The material density was calculated by weighting a regular-shaped sample (with well-defined dimensions and volume) on a precision scale and dividing the mass by the volume. The thermal conductivity, κ, is calculated as κ = α⋅ρ⋅C_p_, with α, ρ, and C_P_ being the thermal diffusivity, bulk density, and specific heat, respectively (Table S3). To confirm the method, the thermal diffusivity and thermal conductivity of drop-cast thick films of formulation 0 (R) and 0* (without ethanol vapor post-treatment) were measured. The thermal diffusivity values were 0.147 ± 0.001 and 0.137 ± 0.001, respectively, and the thermal conductivity values were 0.403 ± 0.002 and 0.367 ± 0.004, respectively. These values align with the thermal diffusivity and conductivity values reported in the literature^[4,5]^.

**Aerogel printing on a stretchable substrate.** The prepared PEDOT:PSS based hydrogel was printed with an in-house modified DIW 3D printer(modification inspired by Krige et al.^[6]^) on a silicone-based elastomer substrate (Ecoflex™ 00-30, Smooth-on) with an average extrusion speed of 0.5 mm/s and feed rate of 30 mm/min. To increase the adhesion between the substrate and the printed geometries, a semi-cured (mixed with crosslinker but not heat treated) Ecoflex thin film was coated on top of the fully cured substrate. Afterward, a PLA frame was placed on the substrate and around the printed features to avoid wrinkling during the subsequent liquid nitrogen dipping and freeze-drying (in a freeze-drier Alpha 2-4 LCSbasic, Martin Christ) steps. We ensured the homogeneity of the aerogel structure during lyophilization by using rapid freezing with liquid nitrogen (LN) on relatively small samples. This method minimizes uneven ice crystal formation and facilitates fast, non-directional ice and pore formation. To complete the Ecoflex substrate's curing and the GOPS crosslinking in the aerogel, the 3D printed device was kept on a hot plate at 120°C for an hour after freeze-drying. The devices’ solvent annealing post-treatment was carried out by keeping them next to an open vial with ethanol in a desiccator for 6 hours under vacuum conditions. This was followed by heat treatment on a hot plate at 120°C for an hour in an ambient atmosphere.

For the stretchable conducting lines test and the planar thermoelectric demos, the 3D printed arches were interconnected/coupled with stretchable conductive silver ink (DM-SIP-2006, DYCOTEC). The silver ink was dried at room temperature overnight, followed by hot-plate annealing at 60°C for 30 minutes in ambient conditions. In some stretchable demos, a surface mounted device (SMD) LED was connected to the circuit. The stretchability of the demos was tested with an in-house-made stretching setup consisting of two clamps in which separation distance was controlled by rotating a screw. For the thermoelectric characterization of the planar thermoelectric demo, a small Peltier module was assembled in one of the clamps to heat one side of the device.

**Dense vs. aerogel pillar’s output power comparison.** First, a reference DIW paste consisting of a PEDOT:PSS commercial dispersion with 5 %vol of DMSO (labeled as sample 0*) was prepared as mentioned above (non-filtered case without additives). The 0* paste was printed and dried at ambient conditions (dense pillar). The paste 0f (F) was freeze-dried after printing. To achieve the same dry pillar diameter for both pastes, the 0* paste had to be printed with a larger nozzle diameter than the 0f (F) paste to compensate for the substantial shrinkage suffered by 0* during ambient drying. Then, both dry pillars were post-treated by ethanol vapor annealing. For the test setup, a thermally conductive and electrically insulating substrate (RS PRO Thermally Conductive Gap Filler Material, thermal conductivity = 1.6 W/(m K)) was placed on a metal piece sitting on a Peltier unit. Silver paste (DM-SIP-2006, DYCOTEC) was used to attach both pillars to the substrate and to connect silver wires from the top and bottom ends of the pillars for electrical measurements. Different temperatures were applied to the substrate with the Peltier unit. The temperature gradient across the pillars was recorded with a thermal camera (PI640, Optris). The external temperatures applied to the pillar, i.e., the temperature of the substrate and the air above the pillar, were measured with a thermocouple attached to thermal paste on the substrate and a thermometer (Sensirion), respectively. The output power was recorded with a source meter unit (SMU, Keysight B2901B).

**Statistical Analysis.** For measurements with associated error bars, we reported the average of at least three data points. We determined the error using the standard error (standard deviation divided by the square root of the number of measurements). For electrical conductivity and Seebeck coefficient measurements of films and aerogels and mechanical tests of films, we sampled three specimens (or more in multiple cases) from each composition, while each Seebeck coefficient measurement involved recording the thermovoltage at three temperatures (or more in multiple cases) and performing a linear regression (the Seebeck coefficient = the fitted slope). The statistical significance of the effect that Li Salt, GOPS, and post-treatment had on the electrical conductivity and Seebeck coefficient of the films was investigated using JMP software. The multiple regression models were obtained by backward subtraction using F-testing with 5% significance, and the results are shown in Table S2 and Figure S7. In Figures 3c and 3d, we took as the maximum bending strain, the maximum achieved (best) for all the samples belonging to the same population (three tested samples), to account for potential structural imperfections and to test the material's maximum capability. Data in Figures 5 – 7 correspond to measurements performed on a single demo device. Errors reported for the power factor and *ZT* were determined from the errors of the Seebeck coefficient, electrical conductivity, and thermal conductivity using the error propagation theory.

**
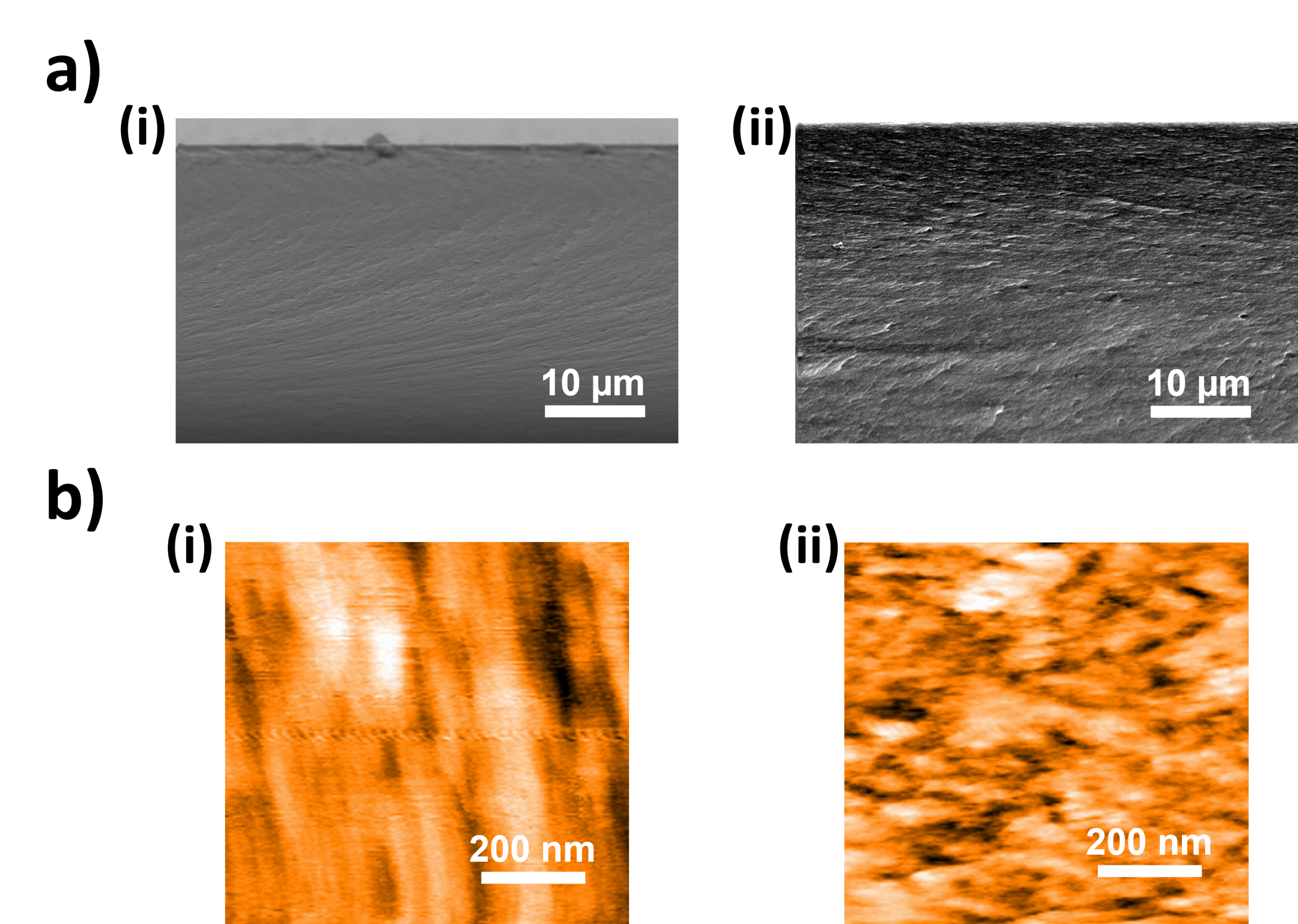
**

**Figure S1. SEM and AFM images of drop-casted PEDOT:PSS films.** a) Cross-section SEM and AFM images b) showing the difference between the pristine (commercial) and filtration-assisted PEDOT:PSS. In both cases, the former shows a smoother morphology than the latter, attributed to an excess of PSS^[7]^.

**
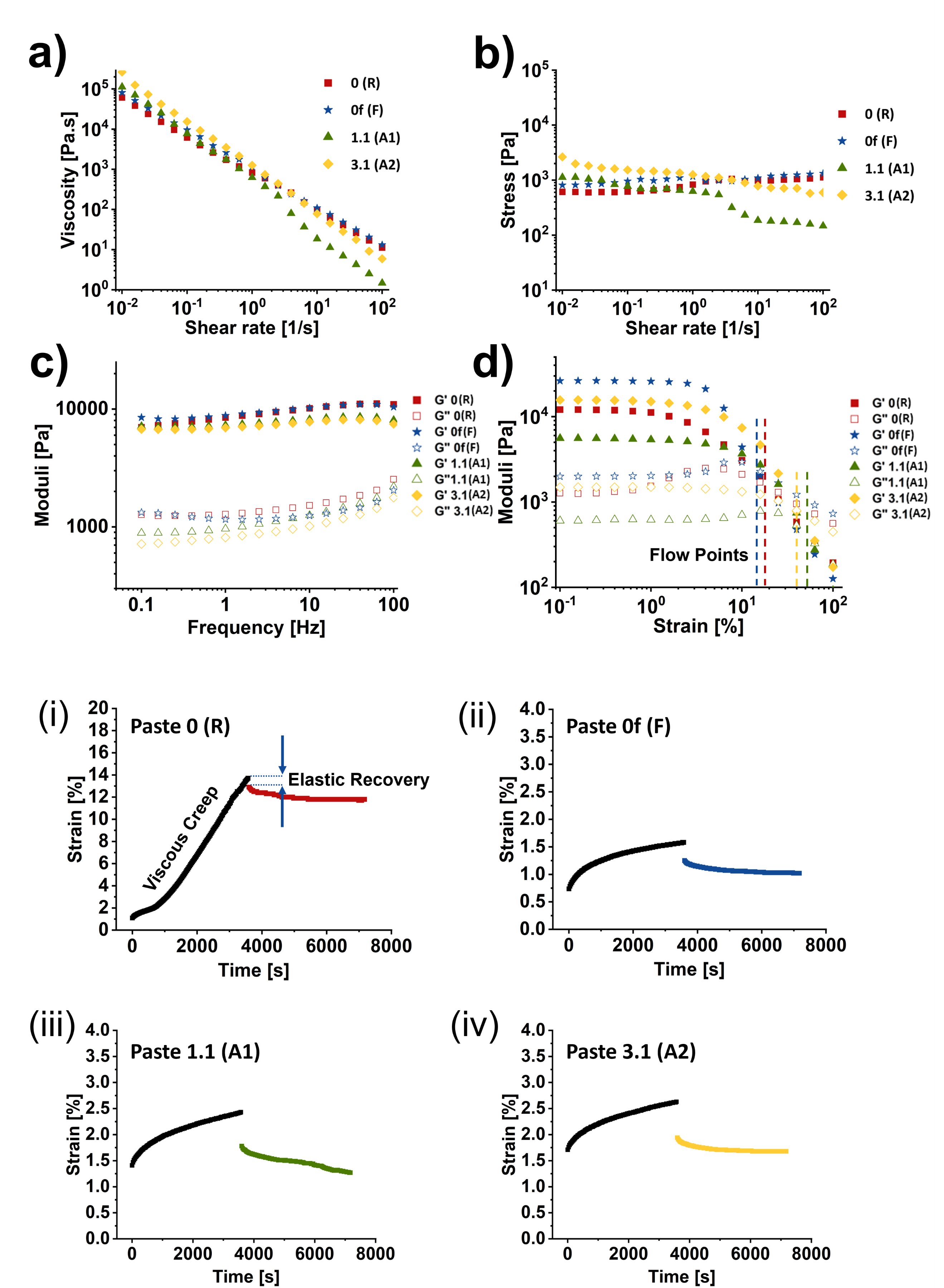
**

**
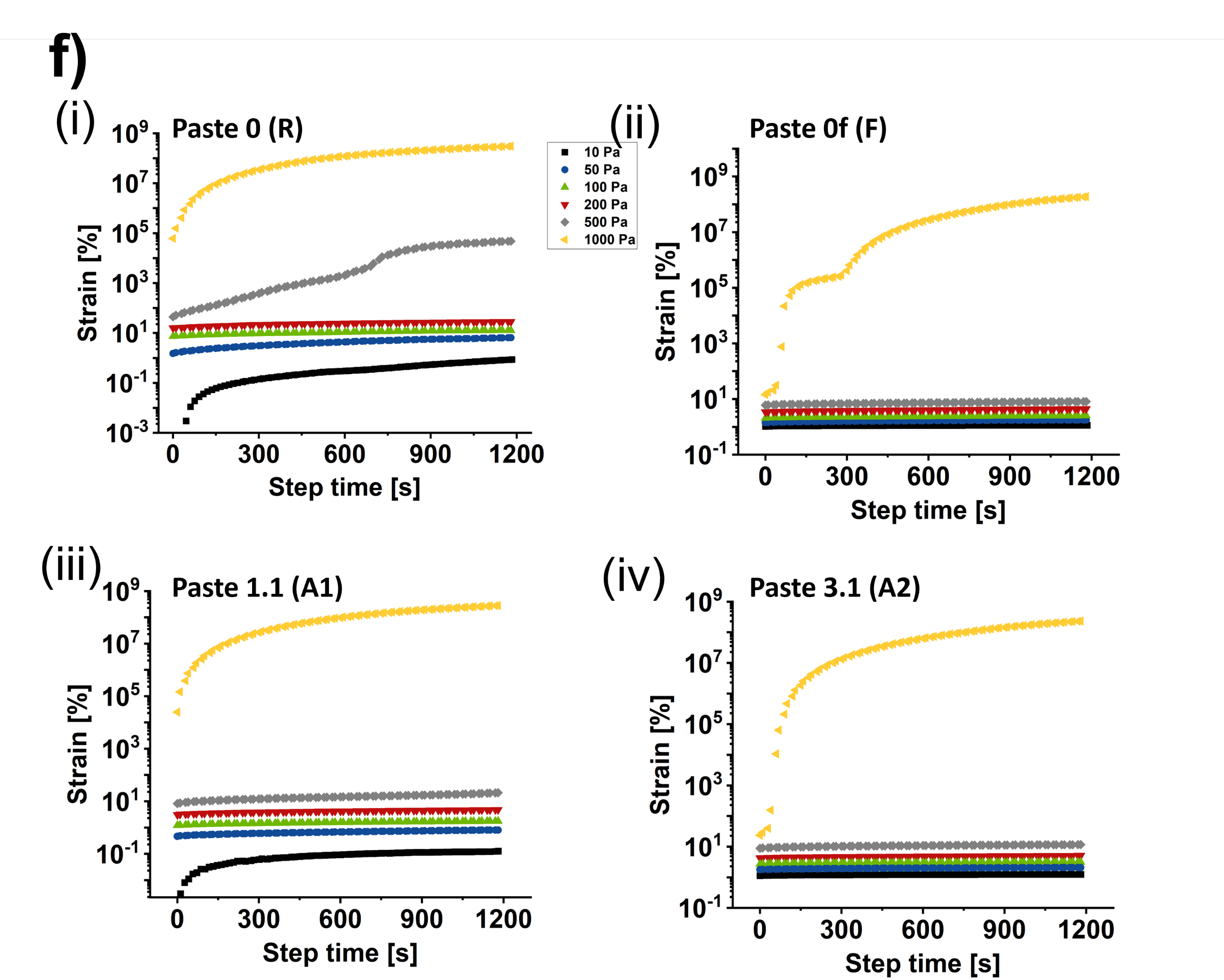
**

**Figure S2. Rheological characterization of selected DIW printable materials.** a) Flow curve. b) Stress change with respect to the applied shear rate. c) Frequency sweep at 1% strain. d) Strain sweep at 10 rad/s. e) Creep-recovery test. f) Stress sweep.

**2. Rheology considerations**

To determine the relevant shear rate range for the flow sweeps (Figure S2a), an order of magnitude estimate of the shear rate at the nozzle wall in the 3D printing process can be obtained from the printing flow rate. For estimation purposes, we assume a constant viscosity and absence of wall slip, which results in a parabolic flow profile in the nozzle with zero velocity at the wall. The flow rate follows from the extrusion velocity, *v*, and plunger surface area, *A*, as:

$$Q=v\cdot A\approx{10}^{-8} m^{3}/s (Eq. S1)$$

Subsequently, the maximum shear rate, which is the shear rate at the wall, can be obtained as:

$\dot{\gamma}=\frac{4Q}{\pi R^{3}}\approx30 1/s (Eq. S2)$

We assume that the DIW paste is a Herschel-Bulkley fluid, with shear stress $\tau$ given by:

$$\tau=\tau_{0}+ k {\gammȧ}^{n} (Eq. S3)$$

This results in the following expression for the viscosity $\eta$:

$$\eta=\frac{\tau}{\dot{\gamma}}=\frac{\tau_{0}}{\dot{\gamma}}+ k {\gammȧ}^{n-1} (Eq. S4)$$

where $\tau$_0_ denotes the yield stress, k stands for the consistency index, $\dot{\gamma}$represents the shear rate, and *n* denotes the flow index. When $\tau$ is smaller than $\tau$_0_, the Herschel-Bulkley fluid exhibits the characteristics of a rigid (non-deformable) solid. Otherwise, it behaves as a fluid. For *n* values less than 1, the fluid demonstrates shear thinning behavior, while for *n* values greater than 1, the fluid exhibits shear thickening behavior. When *n* equals 1 and $\tau$­_0_ equals 0, this model simplifies to that of a Newtonian fluid. As can be observed from Figures S2a and S2b, within the accessible range of shear rates, the slope of the viscosity versus shear rate curve (on a log-log scale) is -1, which means that the flow curve is dominated by the yield stress $\tau_{0}$ around 1000 Pa for all samples (Fig. S2b).

The high yield stress of the hydrogel allows it to withstand deformations of the printed structure due to gravity and surface tension. The surface tension effects, driving retraction of printed filaments, can be quantified by the Laplace pressure:

$\Delta P=\frac{2\gamma}{R}$ (*Eq. S5*)

with the surface tension *γ* ~ 70 mN/m (aqueous paste) and the nozzle radius *R* ~ 800μm, this results in a Laplace pressure $\Delta P$ of $\sim$175 Pa. Thus, the yield stress is largely sufficient to avoid retraction due to the surface tension. The effect of gravity on printability can be determined by the following condition:

$1>\frac{\rho gh}{2\tau_{0}}$ (*Eq. S6*)

where ρ is the density of the material, *g* is the gravity acceleration, and *h* is the height of a virtual column of liquid on top of the material. This dimensionless ratio, which resembles the plastic Galilei number, represents the ratio of the gravitational stress to the yield stress. According to Eq. S6, it is expected that the bottom layers of our aerogel pillars can carry up to a column 0.2 m height of printed material on top before it yields.

Figures S2c and S2d demonstrate the elastic behavior of all hydrogels at low strains, transitioning to more viscous behavior at strains above 10%.

Figure S2e shows the creep-recovery response of the hydrogels, whereby a stress of 50 Pa is applied for 3600 s, followed by recovery under zero stress. During the application of the stress, it can be seen that a large portion of the deformation originates from viscous creep, even below the yield stress/strain. All materials exhibit a similar amount of limited immediate elastic recovery of ~1% when the applied stress is released (downward jump in strain in Figures S2e (i)-(iv) at 3600s). The addition of Li salt (sample *1.1 (A1)* and *3.1 (A2)*) and the freeze drying (sample *0(R)*) make the material less deformable under the application of constant stress below the yield stress while not altering other rheological properties significantly (comparison of the maximal strain in Figures S2e (i) with (ii)-(iv) and rheological results in S2e a, b and c). Figure S2f shows stress sweep tests, with each stress being applied for 1200 s. These confirm that yielding occurs between 500 and 1000 Pa, with sample 0 having a slightly lower yield stress value and more viscous creep at low stress values.


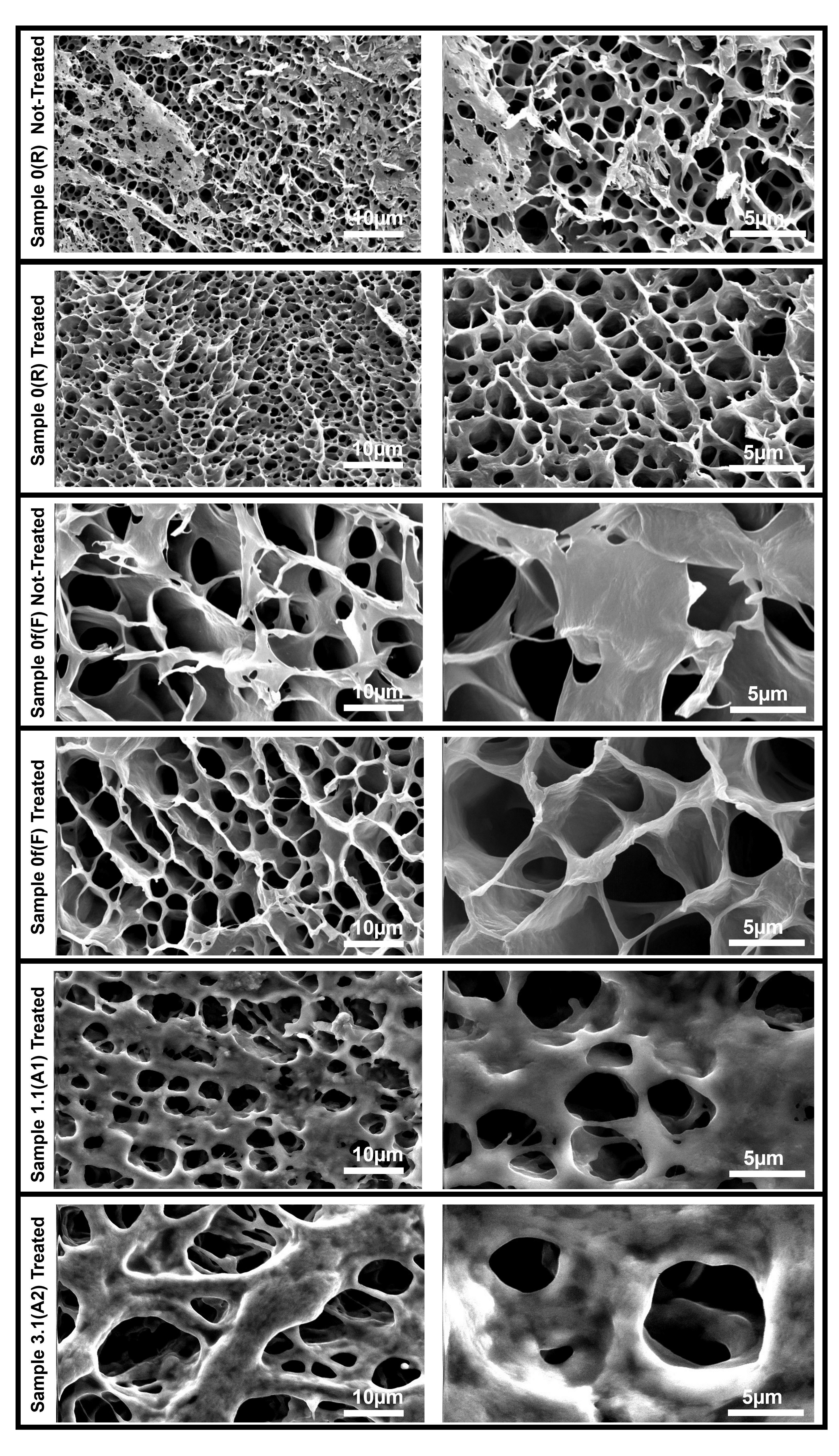


**Figure S3. Scanning Electron Microscopy (SEM) images of the printed aerogels with selected compositions.** The SEM images include both materials before and after ethanol vapor annealing post-treatment. The images reveal that the post-treatment respected the original porosity of the material and improved its surface by welding loose edges (presumably rich in PSS).

**
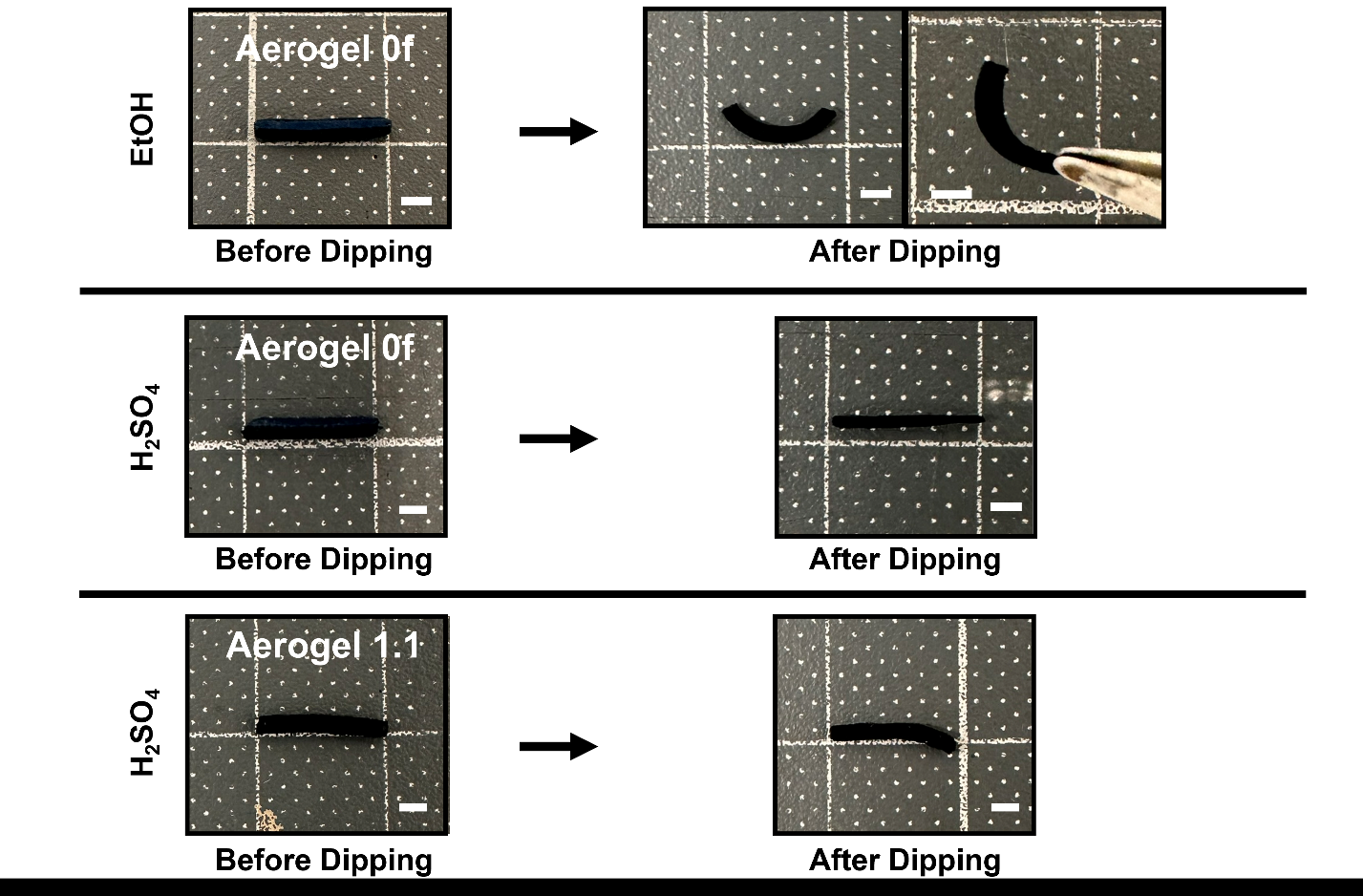
**

**Figure S4. Optical photographs to demonstrate the shrinkage and shape distortion effect of EtOH and H2SO4 dipping post-treatment on different PEDOT:PSS aerogel formulations.**

**Table S1. Material composition and corresponding labeling.** According to the manufacturer of the PEDOT: PSS dispersion (Heraeus Clevios PH1000), the solid content of the dispersion is 1-1.3 wt%. The samples with additives are labeled according to the code x.y, where x = 1-4 represents an increasing (non-proportional) amount of Li salt and y = 0 - 2 represents the amount of GOPS according to Figure S5a. “f” means the filtered formulation. Labeling in parenthesis is used in the main text: R = reference formulation; F = filtered formulation; A1, A2 = formulation with additives.

| **Label** | **Sample Type** | **PEDOT:PSS (ml)** | **DMSO (ml)** | **GOPS (mg)** | **Li Salt (mg)** |
| --- | --- | --- | --- | --- | --- |
| **0 (R)** | Reference | 10 | - | - | - |
| **0*** |  | 10 | 0.2 | - | - |
| **0f (F)** | Filtered | 10 | 16.7  (replaced with water during the filtration) | - | - |
| **0.1** | With Additives | 10 | - | - | 33 ± 3 |
| **0.2** |  | 10 | - | - | 66 ± 3 |
| **0.3** |  | 10 | - | - | 140 ± 5 |
| **0.4** |  | 10 | - | - | 200 ± 5 |
| **1** |  | 10 | - | 6 ± 1 | - |
| **1.1 (A1)** |  | 10 | - | 7 ± 1 | 142 ± 5 |
| **2** |  | 10 | - | 18 ± 1 | - |
| **2.1** |  | 10 | - | 17 ± 1 | 33 ± 3 |
| **2.2** |  | 10 | - | 17 ± 1 | 200 ± 5 |
| **3** |  | 10 | - | 33 ± 3 | - |
| **3.1 (A2)** |  | 10 | - | 28 ± 3 | 140 ± 5 |
| **3.2** |  | 10 | - | 33 ± 3 | 209 ± 5 |


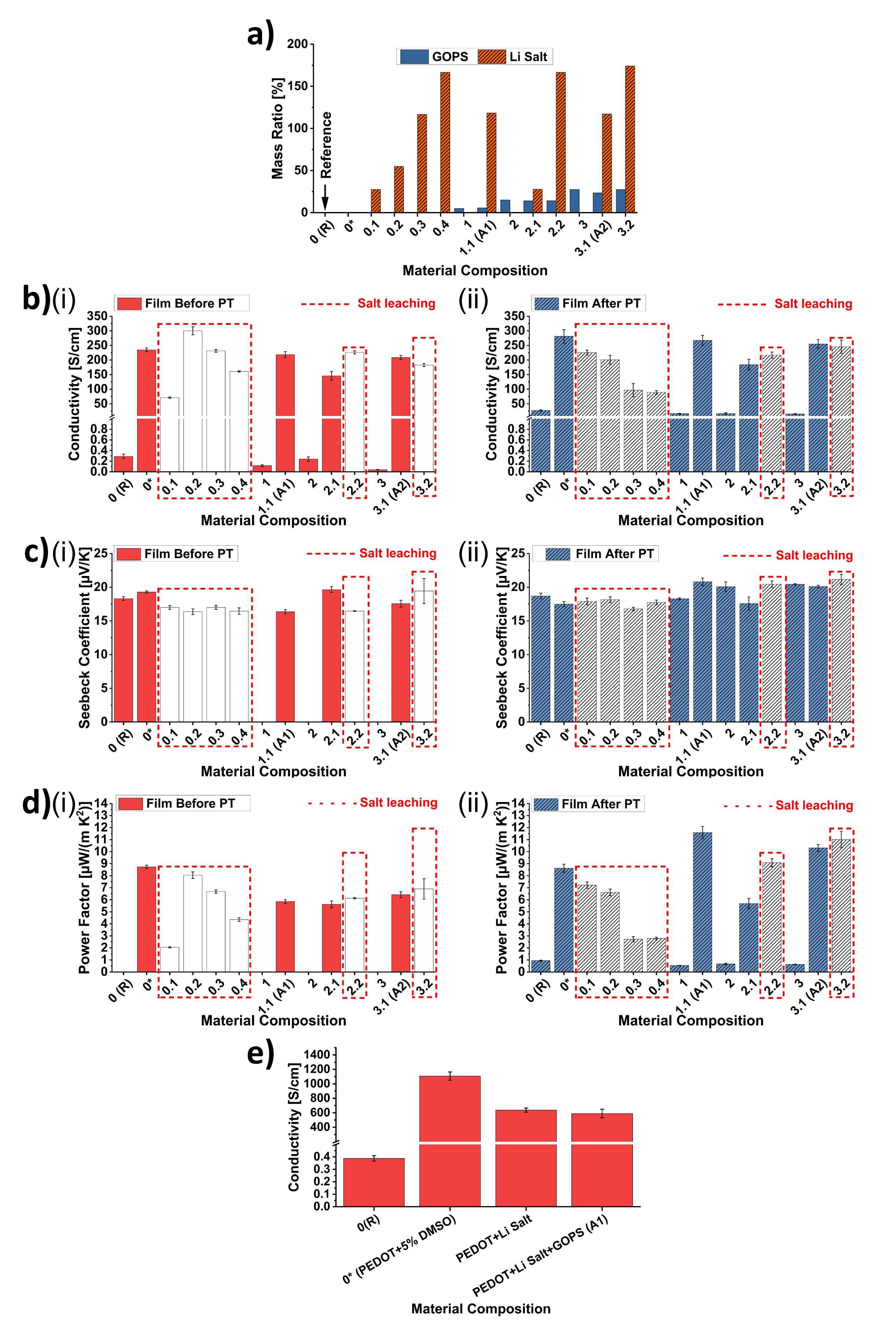


**Figure S5. Thermoelectric characterization of thick and thin PEDOT:PSS films for material composition selection (before and after ethanol vapor annealing post-treatment).** a) Mass ratio of Li salt and GOPS compared to solid PEDOT:PSS (assuming the solid content of PEDOT:PSS in the commercial dispersion is ~ 1.2 wt%) for each tested composition. The sample 0* refers to the benchmark PEDOT:PSS dispersion with 5%vol DMSO. b) Electrical conductivity, c) Seebeck coefficient, and d) power factor for each composition (**the chemically unstable compositions for which salt leaching was observed are marked with red dashed lines and ignored for the material selection**). e) Electrical conductivity of spin-coated PEDOT:PSS thin films (thickness <100 nm) to demonstrate the high conductivity of the base material (not post-treated). All samples were characterized at ambient conditions. For details about material composition, refer to Table S1.


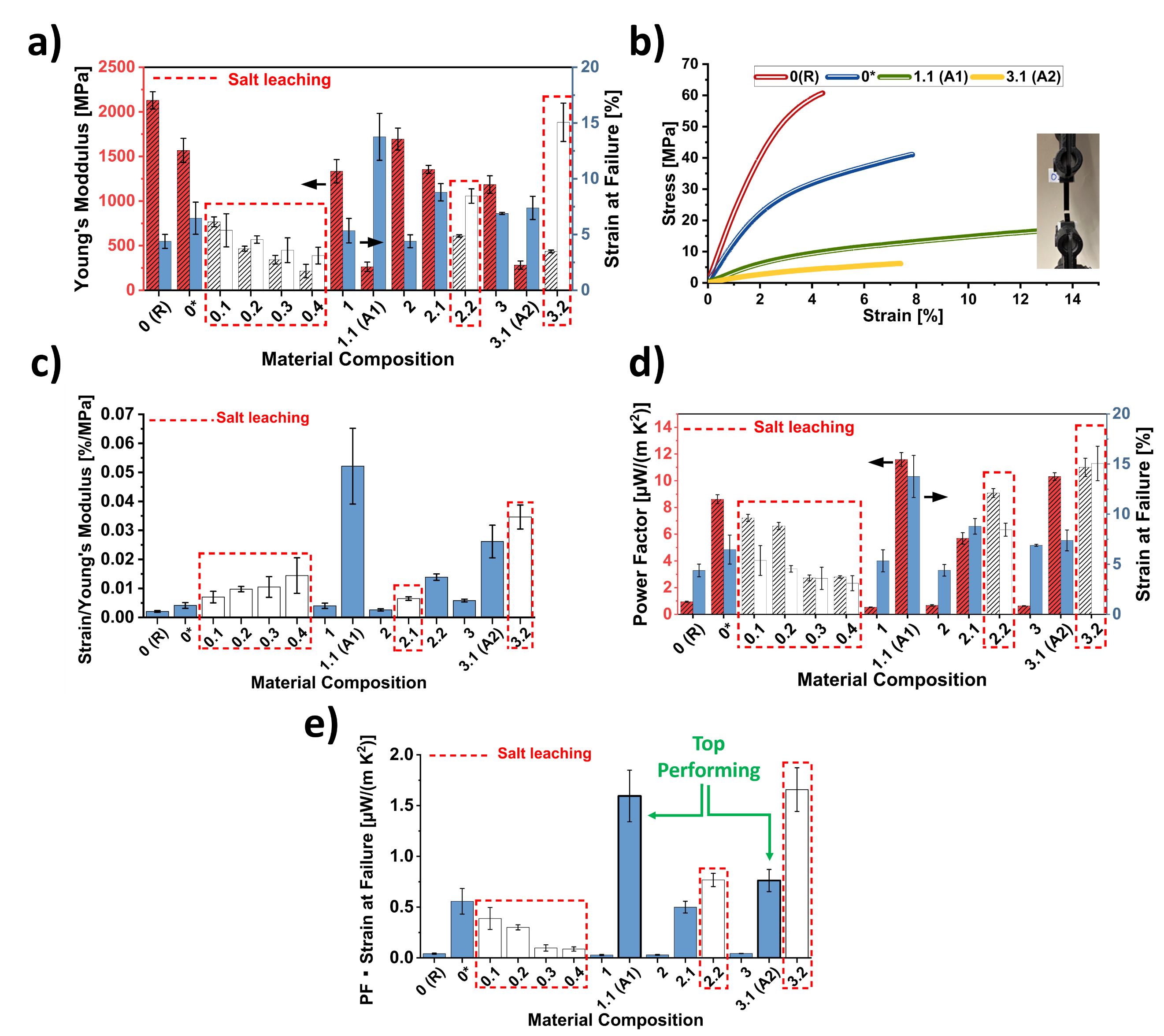


**Figure S6. Mechanical characterization of thick PEDOT:PSS films for material selection.** a) Young’s modulus and strain at failure. b) Representative stress-strain curves. c) Ratio of strain at failure to Young’s modulus. d) Power factor and strain at failure, and e) electromechanical figure of merit (power factor ∙ strain at failure). All samples were post-treated via ethanol vapor annealing and characterized at ambient conditions. **The unstable samples that suffer from salt leaching due to the high salt content and insufficient GOPS are marked with red dashed lines and ignored for the material selection**.

**3. Effect of the additives on thermoelectric and mechanical performance on thick films and material selection for aerogels**

To understand the influence of the addition of Li salt (plasticizer) and GOPS (crosslinker) on the mechanical and thermoelectric properties of PEDOT:PSS thick films, different formulations were developed, as depicted in Figure S5a. and Table S1. Pristine PEDOT:PSS without additives (formulation *0 (R)*) and the widely-reported high-conducting PEDOT:PSS with 5%vol DMSO^[8,9]^ (formulation *0**) were taken as references. Although the electrical conductivity of *0** exceeded 1100 S/cm when spin-coated as thin films (Figure S5e), the value for thick films was only 280 S/cm (Figure S5b). Both the conductivity values and the behavior of conductivity decreasing with film thickness are consistent with previous reports^[5,10]^. As mentioned in reference^[5]^, the cause of this difference is unclear. Still, the possible explanation given by the literature is that variations in evaporation temperature and time during the preparation of thick and spin-coated films could impact the molecular-scale kinetics, thus influencing the polymer morphology. This hypothesis aligns with previous studies that showed a significant impact of evaporation temperature on the electrical conductivity of DMSO-mixed PEDOT films^[11,12]^. In any case, the high conductivity measured for thin films demonstrates that the aerogels’ performance is not constrained by an unoptimized base material.

It is well-known that dipping PEDOT:PSS films in a polar solvent or acid followed by a washing step further improves electrical conductivity^[13]^. However, dipping causes substantial shrinkage and shape distortion in aerogels during the post-dipping drying phase (Figure S4). Since shape retention is essential for 3D-printed structures, dipping is not considered in this work. Instead, the samples were subjected to ethanol vapor annealing post-treatment, which significantly enhanced the power factor by boosting markedly the electrical conductivity and, to a lesser extent, the Seebeck coefficient (Figure S5 and Figure S7). Vapor annealing with a polar solvent has been reported as an alternative strategy to realize secondary doping leading to an improved conductivity^[14]^. Figure S5b and Figure S5c show the room-temperature electrical conductivity and Seebeck coefficient of the post-treated films made from the different formulations. While material composition showed only a moderate effect on the Seebeck coefficient (ranging from 18 – 21 μV/K), the electrical conductivity increased dramatically with the addition of small amounts of Li salt from 27 ± 4 S/cm for pristine PEDOT:PSS (sample *0(R)*) to 225 – 275 S/cm for samples *0.1*, *1.1 (A1)* and *3.1 (A2)* (for comparison equivalent thin films achieved conductivity up to 600 S/cm, consistent to values reported in the literature^[15]^). These high electrical conductivity values are comparable to those measured for the high-conductivity benchmark PEDOT:PSS with 5%vol DMSO. However, excessive Li salt content resulted in the salt leaching out of the material (marked with the red dashed frames on the graphs), which led to instability and eventual conductivity degradation. The statistical significance of Li salt addition and leaching in the conductivity has been confirmed in Figure S7. Interestingly, the addition of GOPS alone showed little influence on the electrical conductivity. Yet, it promoted the assimilation of Li salt in the PEDOT:PSS, pushing up the maximum stable (without leaching) loading. This synergy between Li salt and GOPS was also statistically confirmed (Figure S7) and resulted in a maximum power factor of 11.6 ± 0.5 μW/(m K^2^) for the stable composition *1.1 (A1)* after ethanol vapor post-treatments. Regarding mechanical performance, in the absence of GOPS, Young’s modulus values decreased with Li salt, while the strain at failure increased (samples *0(R)* and *0.1* to *0.4* in Figure S6a, Figure S6c, and Figure S6d). Adding GOPS alone (samples *1*, *2.0*, *3.0*) showed no clear trend in Young’s modulus but slightly increased the strain at failure. Hence, similar to what we observed for the electrical properties, the addition of both GOPS and Li salt contributed to boosting mechanical properties. In particular, we measured an increase of strain at failure from 4.4 ± 0.6 % for pristine PEDOT:PSS (sample 0(R)) to 14 ± 2 % for sample *1.1 (A1)*. However, it is worth noting that contrary to what we observed for the power factor, the positive effect of additives on the material's mechanical properties seems to be independent, with no statistically significant synergy between additives being identified (Figure S7). To reflect Li salt's and GOPS's electromechanical benefits, we propose a new combined property, the electromechanical figure of merit (*PF* ∙ strain at failure) (Figure S6e). According to this electromechanical figure of merit and the samples’ salt leaching stability, formulations *1.1* and *3.1* were selected as the most promising materials to make aerogels. In the main text, these formulations are called A1 and A2.

**4. Significant effect of the additives**


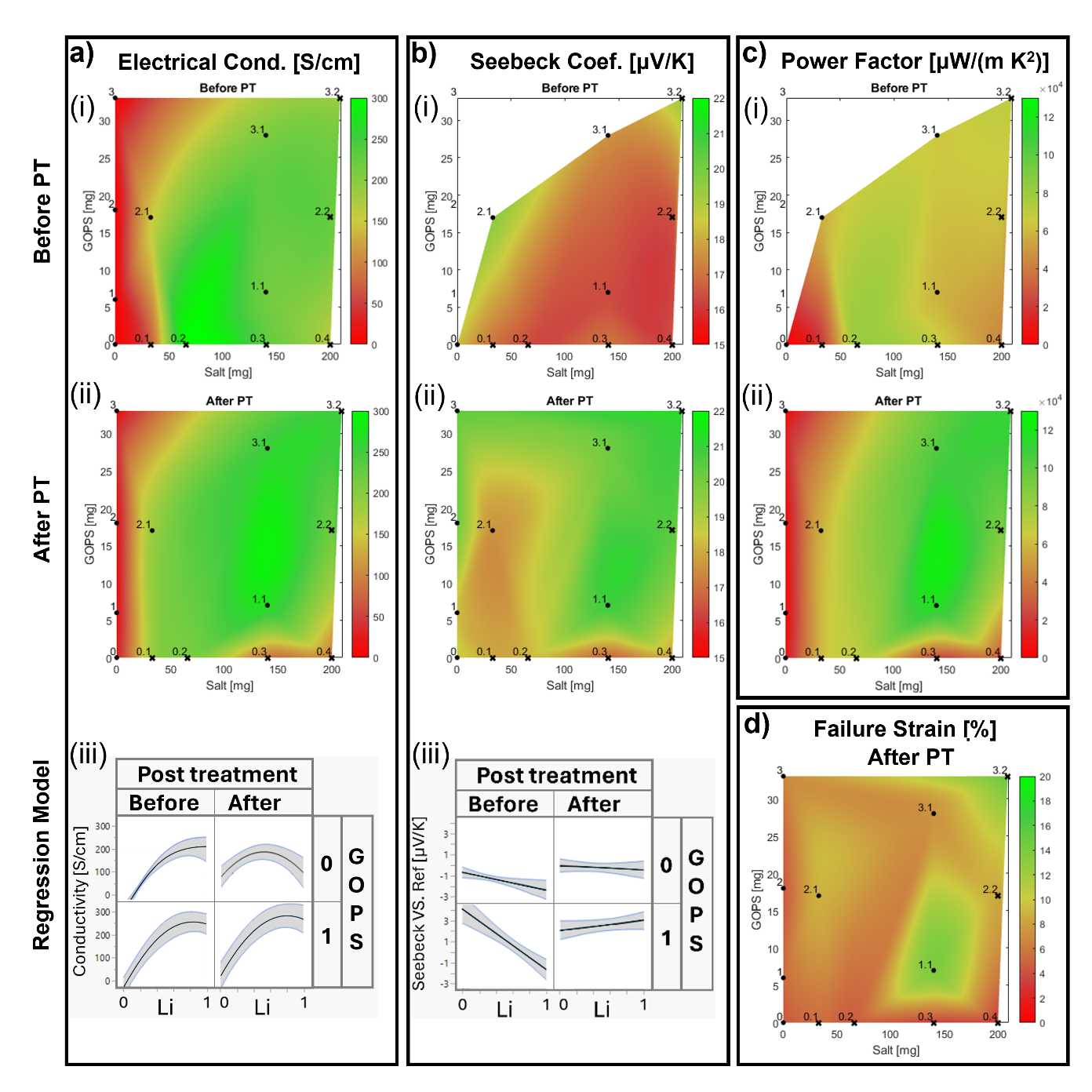


**Figure S7. Thermoelectric and mechanical performance color plots and statistical analysis for thick films of PEDOT:PSS with varying Li salt and GOPS concentrations.** a) Color plot depicting conductivity before (i) and after (ii) post-treatment (PT) and the regression model profiles (iii). b) Color plot depicting Seebeck coefficient before (i) and after (ii) post-treatment and the regression model profiles (iii). c) Color plot depicting power factor before (i) and after (ii) post-treatment (PT). d) Color plot depicting failure strain after post-treatment (PT). The color plot utilizes cubic interpolation to smooth the discrete data from Figure S5 and S5’s data. Each formulation is marked in the design space by the marker “•” (or “**x**” in the case of Li salt leaching). In the regression model profile plots, the maximum and minimum amounts of Li salt and GOPS are indicated by 1 and 0, respectively.

To evaluate the statistical significance of the effects of Li salt, GOPS, and post-treatment on the electrical conductivity and Seebeck coefficient of thick films, a multivariable regression based on backward subtraction (F-test, significance level of 5%) was employed. The variables “amount of Li salt” and “amount of GOPS” were coded from 0 to 1 so that no interaction is possible when one of the additives is absent to reflect physical conditions accurately. The pristine PEDOT:PSS film (formulation *0 (R)*) without post-treatment with all variables at the low level served as the reference and was subtracted from all others, effectively excluding the intercept term from the analysis.

**Conductivity (*σ*):** The response surface in Figure S7a and the regression model in Table S2 indicate that there is a strong and positive influence of Li salt on conductivity (coefficient = 326), reaching a maximum value before declining due to the detrimental effect of salt leaching at high salt mass ratio. Note that leaching leads physically to an actual decrease in the amount of salt within the material. This quadratic Li salt mass ratio and conductivity trend are underpinned by the statistically significant quadratic Li*Li effect (P < 0.0001) in Table S2. While GOPS exhibited a statistically significant effect (P = 0.035), it was comparatively more minor (coefficient = 50) than Li and Li*Li. Post-treatment (PT) also exhibited a significant and slightly positive effect on conductivity (coefficient = 38, P = 0.0106). Even though there was a clear negative influence on the conductivity of combining post-treatment with the use of Li salt (PT*Li, coefficient = -172, P < 0.0001), the further addition of GOPS led to a clear positive synergy (PT*Li*GOPS, coefficient = 227, P = 0.0029). This synergy is evident in Figure S7a, where post-treatment induced a shift in the optimal region of conductivity towards higher Li salt and GOPS amounts.

In summary, the regression analysis and color plot indicate that while Li salt greatly benefits conductivity, it is susceptible to leaching, particularly in large concentrations and during post-treatment. GOPS alone offers a minor direct benefit but synergizes with Li salt by stabilizing it, leading to optimal performance at elevated levels of both Li salt and GOPS. This synergy requires ethanol vapor post-treatment.

**Seebeck coefficient (*S*):** Figure S7b and Table S2 illustrate the limited, albeit statistically significant (P < 1% in all cases) effect of Li salt, GOPS, and PT on the Seebeck coefficient. Post-treatment is found to be crucial to turn the adverse impact of Li salt and the interaction Li*GOPS (coefficients = -3.1 and -4.2, respectively) into a positive three-way PT*Li*GOPS synergy (coefficient = 5.5).

**Power factor:** Consistent with the previous analysis, Figure S7c and Table S2 illustrate that the highest power factor (calculated as *S*²∙conductivity) is achieved in films that have undergone post-treatment, contain high levels of Li salt and GOPS, and do not display salt leaching.

**Mechanical—Failure Strain (only for films after PT):** The color plot (Figure S7d) and regression analysis (Table S2) for the failure strain of films after PT did not reveal significant correlations or second-order interactions, only a main positive effect from both Li salt and GOPS.

| **Electrical Conductivity** | | | | **Seebeck Coefficient** | | | | **Failure Strain** | | | |
| --- | --- | --- | --- | --- | --- | --- | --- | --- | --- | --- | --- |
| **Effect (DoF = 68)** | **F Ratio** | **Coef.** | **P** | **Effect (DoF = 58)** | **F Ratio** | **Coeff.** | **P** | **Effect (DoF = 68)** | **F Ratio** | **Coeff.** | **P** |
| Li | 151.3 | 326 | <0.0001 | Li | 85.2 | -3.1 | <0.0001 | Li | 10.4 | 3.9 | 0.0020 |
| Li*Li | 23.5 | - 409 | <0.0001 | GOPS | 64.1 | 2.8 | <0.0001 | GOPS | 4.2 | 2.6 | 0.0448 |
| GOPS | 4.6 | 50 | 0.0350 | Li*GOPS | 7.2 | -4.2 | 0.0095 |  |  |  |  |
| PT | 6.9 | 38 | 0.0106 | PT | 30.9 | 1.2 | <0.0001 |  |  |  |  |
| PT*Li | 20.8 | - 172 | <0.0001 | PT*Li | 37.0 | 3.2 | <0.0001 |  |  |  |  |
| PT*Li*GOPS | 9.5 | 227 | 0.0029 | PT*Li*GOPS | 8.8 | 5.5 | 0.0043 |  |  |  |  |

**Table S2. Statistical analysis of the effects of additives and post-treatment on the thermoelectric and mechanical performance of thick film PEDOT:PSS.** The study includes the effect of Li Salt (Li), GOPS, post-treatment (PT), and their combinations on electrical conductivity, Seebeck coefficient, and failure strain. (DoF: degree of freedom).


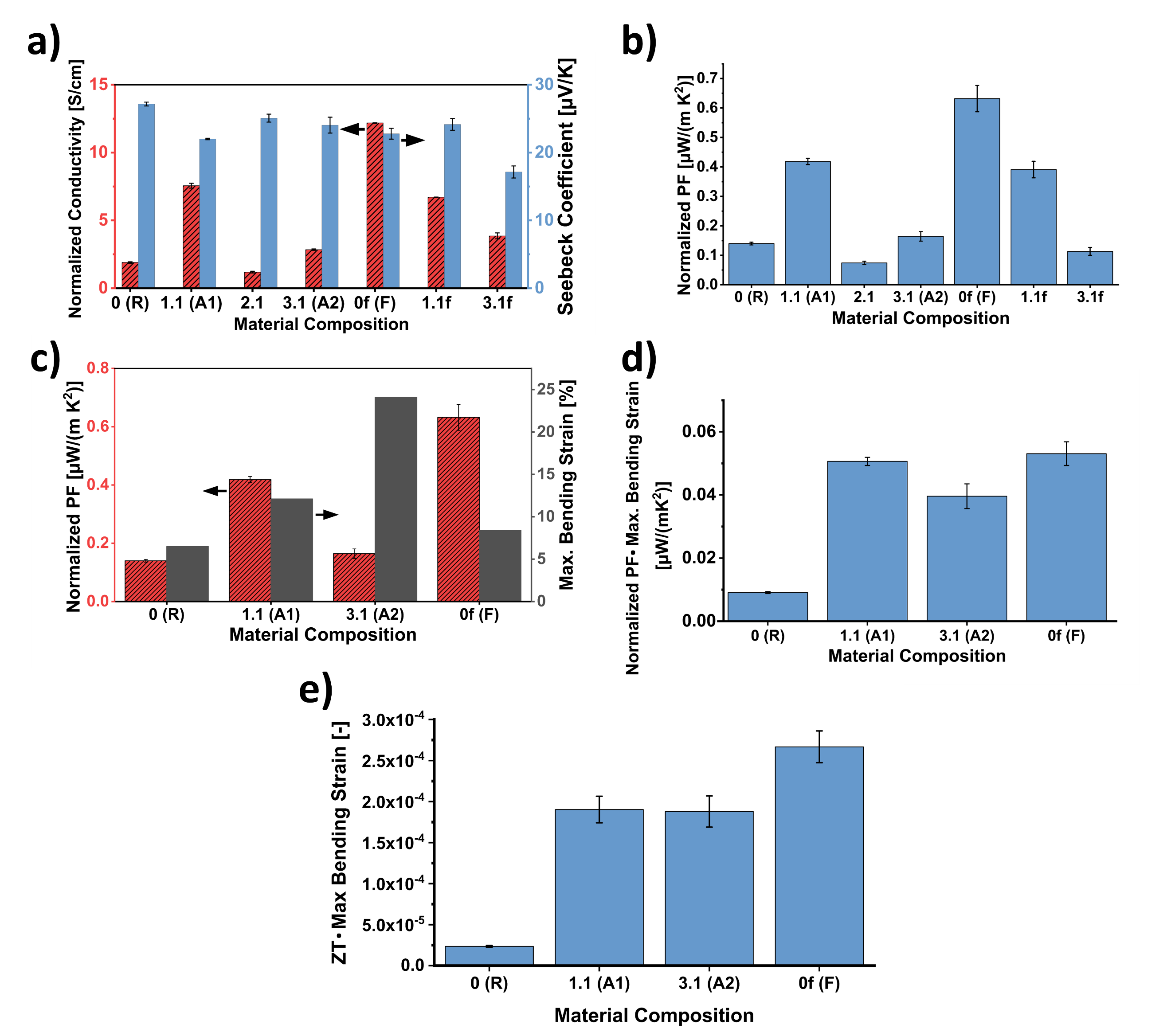


**Figure S8. Normalized (to density corresponding to 90%vol of air) thermoelectrical and mechanical characterization of 3D printed aerogel filaments for material selection.** a) Normalized electrical conductivity and Seebeck coefficient. b) Normalized power factor. c) Normalized power factor and maximum bending strain to which the parts survive upon bending. New electromechanical figures of merit: d) product of normalized power factor and the maximum bending strain, and e) product of ZT and the maximum bending strain. The thermal conductivity was extracted from Table S3. All samples were post-treated via ethanol vapor annealing and characterized at ambient conditions.

**5. Explanation of the conductivity normalization to the aerogel expected density**

The density normalization was employed to adjust for the varying volume fraction of air in each aerogel sample, which arose from the poor control of the total water content in the paste before the freeze-drying step. As a result, the final material density varied significantly for each material composition and slightly for different batches of the same composition (Table S3). To mitigate this variability and enable a fairer comparison among aerogels, we utilized the deviation of each material from its expected density to compensate for conductivity. The expected density was calculated assuming that 90% of the aerogel volume was air, roughly for most measured aerogels. Consequently, materials with a higher density than expected (indicating a comparatively higher ratio of dense phase) were penalized accordingly in terms of conductivity. A similar correction was reported before by other groups in a similar context^[16]^.

The justification for this normalization is the following. We assume the aerogel to be a composite of a (percolated) conducting phase (polymer with conductivity *σ_polymer_* ) and an insulating phase (air with conductivity *σ_air_* ~ 0). Thus, the aerogel conductivity should follow a general rule of mixture^[17]^:

$\sigma_{aerogel}^{\alpha}=f\sigma_{PEDOT:PSS}^{\alpha}+\left( 1-f \right)\sigma_{air}^{\alpha}\approx f\sigma_{PEDOT:PSS}^{\alpha} (Eq. S7)$

where *f* is the volume fraction of the polymer and -1<α<1 is a fitting parameter that depends on the composite morphology. Assuming *α* =1, true only for polymer fibers perfectly parallel to the electric field, Eq. S7 establishes that the aerogel conductivity is proportional to the polymer volume fraction. Likewise, the aerogel density (ρ_aerogel_) is also proportional to the polymer volume fraction: *ρ_aerogel_ ~ f ρ_polymer_*. Hence, *σ_aerogel_* $\propto$ *ρ_aerogel_*.

Although this normalization involves a strong assumption in the aerogel morphology (all polymer fibers parallel to transport) and likely introduces some degree of error, it was useful for a rough qualitative analysis. Indeed, after density correction, a similar trend to that observed for films in Figure S5 was noted for the aerogels in Figure S8. Note that density normalization was done exclusively for material screening, and no claims of absolute performance were made based on normalized values. The results shown in Figure 3 correspond to actual (non-normalized) measurements.


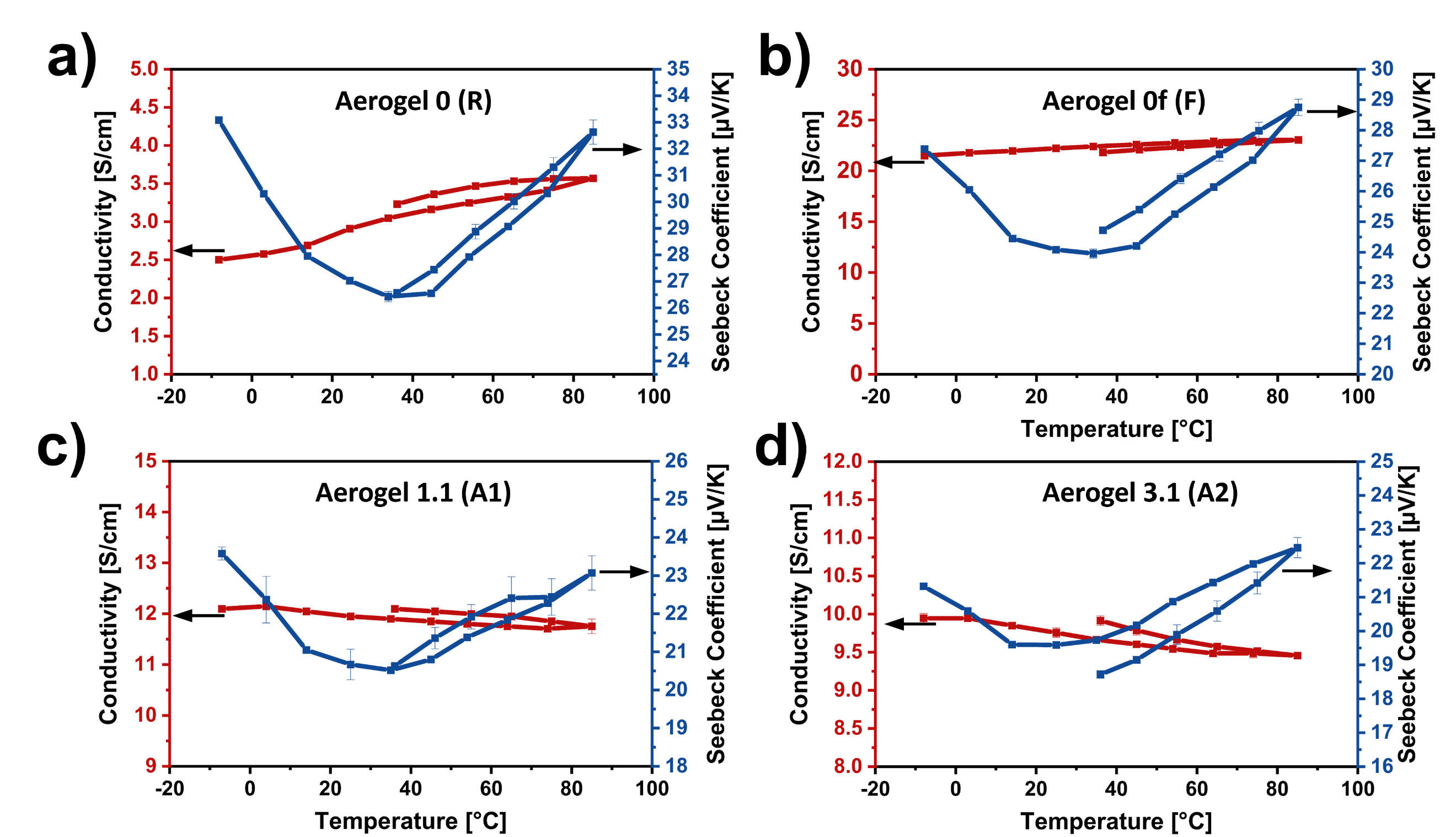


**Figure S9. Electrical conductivity and Seebeck coefficient change with temperature (between -10 °C and 90 °C) for different PEDOT:PSS aerogels.** a) Sample 0 (R). b) Sample 0f (F). c) Sample 1.1 (A1). d) Sample 3.1 (A2). The tested range of temperature is relevant for wearables and applications related to the IoT.

**Table S3. Parameters used for thermal conductivity calculation.**

| **Aerogel** | **Density [g/cm^3^]** | **Average Pore Diameter (μm)** | **Diffusivity [mm^2^/s]** | **Cp [J/(g K)]** | **Conductivity [W/(m K)]** |
| --- | --- | --- | --- | --- | --- |
| 0 (R) | 0.103 ± 0.002 | 1.2 ± 0.1 | 0.746 ± 0.002 | 1.80 | 0.1397 ± 0.0013 |
| 0f (F) | 0.110 ± 0.002 | 4.1 ± 0.2 | 0.358 ± 0.005 | 1.65 | 0.0651 ± 0.0008 |
| 1.1 (A1) | 0.280 ± 0.004 | 3.0 ± 0.2 | 0.328 ± 0.003 | 1.84 | 0.1695 ± 0.0016 |
| 3.1 (A2) | 0.231 ± 0.002 | 3.3 ± 0.3 | 0.286 ± 0.002 | 1.79 | 0.1186 ± 0.0007 |

**6. Aerogel thermal conductivity considerations**

The thermal conductivity, *κ*, is calculated as *κ = α⋅ρ⋅Cp*, with *α*, *ρ*, and *C_P_* being the thermal diffusivity, bulk density, and specific heat, respectively (Table S3).

$$\kappa= \alpha\cdot\rho\cdot Cp (Eq. S8)$$

In traditional metals, where free electrons are the primary contributors of heat and charge, *κ~ κ_e_*. The Wiedemann-Franz law states that the ratio of electronic thermal conductivity (*κ_e_*) to electrical conductivity (*σ*) is proportional to the absolute temperature (*T*), with the proportionality constant being the Lorenz number (*L*).

$$\kappa_{e}= \sigma LT \left( Eq. S9 \right)$$

However, in conducting polymers, the contribution of phonons (quantized lattice vibrations) to thermal conductivity (*κ_p_*) cannot be neglected, leading to:

$$\kappa=\kappa_{p}+\kappa_{e}= \kappa_{p}+ \sigma LT (Eq. S10)$$

According to Table S3, comparing directly *0 (R)* and *0f (F)* is fair as they have similar densities. Interestingly, *0f (F)* has lower thermal conductivity than *0 (R)* despite having higher electrical conductivity. Hence, from eq. S10, we can conclude that *0f (F)* has a much lower phonon contribution to the thermal conductivity than *0 (R)*. *0f (F)* has a lower PSS content than *0 (R)* due to the filtration, which indicates that PSS contributes to the phonon conductivity.

Due to their different formulations, aerogels, including additives, have different rheological characteristics than *0 (R)* and *0f (F)*. To standardize the paste handling during processing, the same consistency and texture were sought for all the different formulations during the paste preparation steps. As a result, pastes with additives end up with comparatively higher concentrations, leading to higher-density aerogels. This is why *0f (F)* has a lower thermal conductivity than the samples with additives (eq. S9).

To shed light on the effect of the additives on the thermal conductivity of the aerogels, we must compare the net effect of the dense phase of the aerogels, which requires the application of the normalization used in section 5. The normalized phonon contribution (κ_p_) to the thermal conductivity for *0 (R)*, *0f (F)*, *1.1 (A1)*, and *3.1 (A2)* are 0.113 ± 0.003, 0.050 ± 0.007, 0.07 ± 0.01 and 0.06 ± 0.001 W/m K, respectively. The neat reference *0 (R)* sample has the highest phonon contribution of all aerogels, indicating that both PSS removal and using additives are valid strategies to decrease phonon thermal conductivity.

It is worth noting that the validity of the discussion above relies on the fact that all our samples possess large and similar pore sizes (see Table S3). This remark is important because it has been reported that the pore size impacts thermal conductivity, especially when the pore size is comparable to the mean free path of phonons in the material, which corresponds to 10’s of nanometers^[18]^. Our material pore size is a few micrometers (Table S3), which is much larger than the mean free path of phonons. At that scale, thermal conductivity should be relatively independent of the pore size. In particular, the optimal pore size to minimize thermal conductivity should be below the mean free path^[18]^. Such small pore size has been achieved before with supercritical drying based on liquid carbon dioxide^[19]^, not with the lyophilization process based on liquid nitrogen we used in this paper.

**
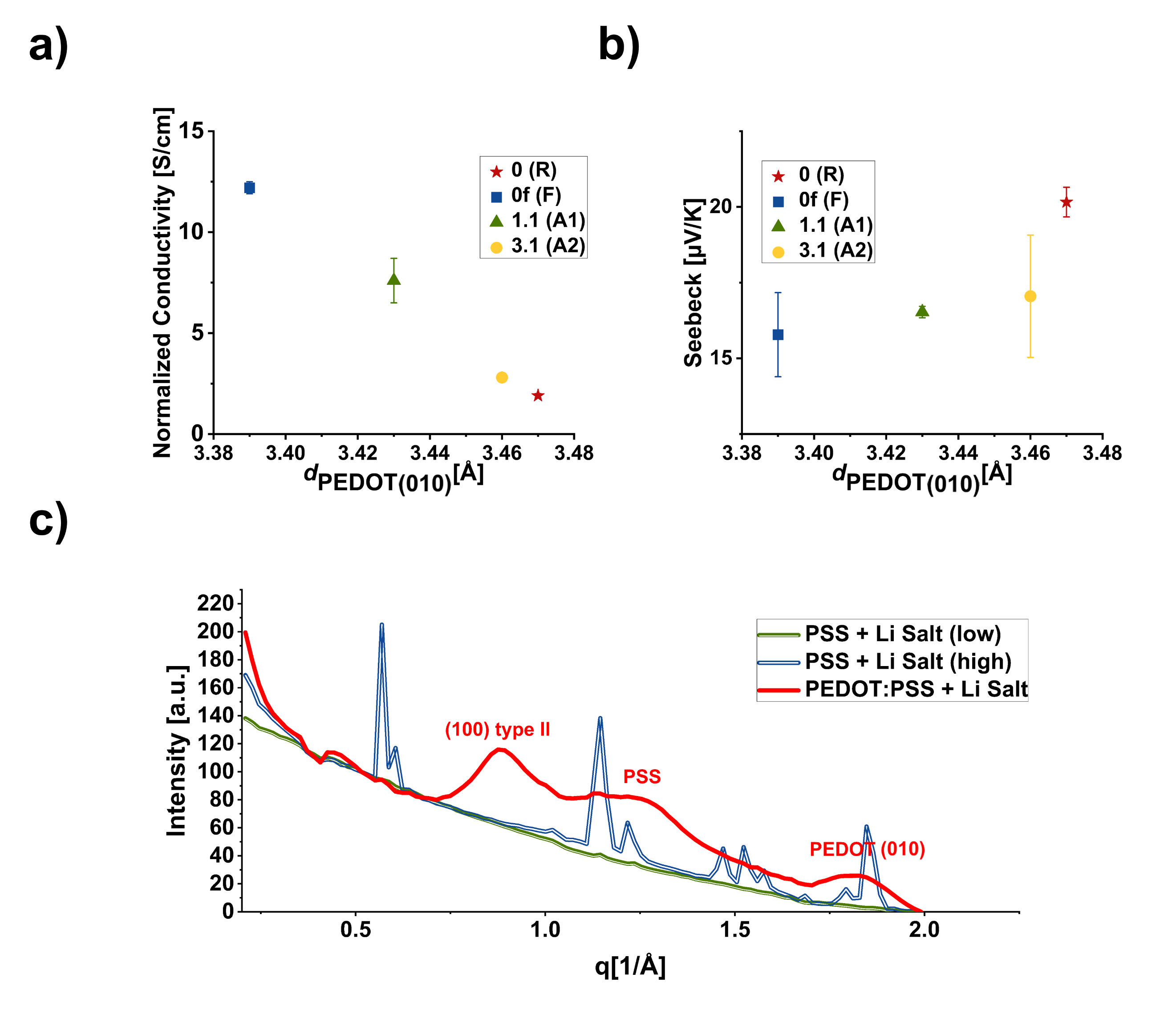
**

**Figure S10. Synchrotron X-ray diffraction study.** a) Electrical conductivity, and b) Seebeck coefficient versus PEDOT π–π stacking distance -peak (010)- measured with Wide-Angle X-ray Scattering (WAXS) in transmission mode. c) Grazing-incidence WAXS (GIWAXS) 1D line-cut intensity profile of PEDOT:PSS with Li salt films and bare PSS with Li salt films (taken as reference).

**Table S4*.* WAXS analysis results for four different PEDOT aerogel samples.** The table shows the q values and d-spacing corresponding to the 100 and 010 peaks of PEDOT and PSS. These peaks provide insights into the structural organization and intermolecular spacing within each aerogel sample, highlighting crystallinity and phase structure variations due to different compositions.

|  | $\boldsymbol{q}_{\boldsymbol{PEDOT}\left( \boldsymbol{100} \right)}$  **(Å^-1^)** | $\boldsymbol{d}_{\boldsymbol{PEDOT}\left( \boldsymbol{100} \right)}$  **(Å)** | $\boldsymbol{q}_{\boldsymbol{PSS}}$  **(Å^-1^)** | $\boldsymbol{d}_{\boldsymbol{PSS}}$  **(Å)** | $\boldsymbol{q}_{\boldsymbol{PEDOT}\left( \boldsymbol{010} \right)}$  **(Å^-1^)** | $\boldsymbol{d}_{\boldsymbol{PEDOT}\left( \boldsymbol{010} \right)}$  **(Å)** | $\boldsymbol{I}_{\boldsymbol{PEDOT}\left( \boldsymbol{010} \right)}$  **(a.u.)** |
| --- | --- | --- | --- | --- | --- | --- | --- |
| **0 (R)** | - | - | 1.27 | 4.93 | 1.81 | 3.47 | 2.45 x10^-3^ |
| **0f (F)** | 0.22 | 28.56 | 1.29 | 4.86 | 1.85 | 3.39 | 3.35 x10^-3^ |
| **1.1 (A1)** | 0.88 | 7.16 | 1.23 | 5.10 | 1.83 | 3.43 | 1.85 x10^-3^ |
| **3.1 (A2)** | 0.88 | 7.13 | 1.25 | 5.04 | 1.82 | 3.46 | 1.60 x10^-3^ |

**7. Thermoelectric output power calculations for the stretchable planar device (aerogel sample # 1.1 (A1))**

**Table S5. Device and material parameters for the stretchable planar device.** The values of conductivity and Seebeck for the aerogel 1.1 (A1) are extracted from Figure 3a. The values of conductivity and Seebeck for the stretchable silver were taken from the manufacturer and measured in our lab, respectively.

| Length of the aerogel leg: | *L* = 6.7 cm |
| --- | --- |
| Aerogel filament cross sectional area (circular): | *A* = 1.5 10^-2^ cm^2^ (radius = 0.14cm) |
| Electrical conductivity of Aerogel *1.1 (A1)* (p-type leg): | *σ_aerogel 1.1_* = 16 S/cm |
| Seebeck coefficient of Aerogel *1.1 (A1)* (p-type leg)∶ | *S_p_* = 23.5 μV/K |
| Electrical conductivity of Silver line (n-type leg): | *σ_silver_* = 2 10^4^ S/cm |
| Seebeck coefficient of Silver line (n-type leg*): | *S_n_* = 6.5 μV/K |

*Even though the silver was used in the n-type leg, it is actually a weak p-type material.

The maximum output power (*P_max_*) provided by a thermocouple harvester is^[20,21]^:

$$\begin{aligned} P_{max}=\frac{V_{OC}^{2}}{4R_{i}}=\frac{\left[ \left( S_{p}-S_{n} \right){\Delta T}_{L} \right]^{2}}{4\left( R_{p}+R_{n}+R_{c} \right)}\#\left( Eq.S11 \right) \end{aligned}$$

Where *V_oc_* is open circuit voltage; *S_p_* and *S_n_* are the Seebeck coefficient of the used p-type and n-type materials, respectively; *ΔT_L_* is the externally-applied temperature difference across the device length; and *R­_i_*, *R_p_*, *R_n_*, *R_c_* are the following electrical resistances: total internal resistance of the thermocouple, resistance of the p-type leg, resistance of the n-type leg, and total contact resistance. Hence, *R­_i_* = *R_p_* + *R_n_ + R_c_*.

The total internal resistance of the device, *R­_i_* = *R_p_* + *R_n_ + R_c_ =* 27.5 Ω, can be extracted from the slope of the measured I-V curve presented in Figure 6b. The resistance of the PEDOT:PSS aerogel p-type leg is, according to the Pouillet’s law:

$R_{p}= \frac{1}{\sigma}\frac{L}{A}=27.3 \Omega(Eq. S12)$

(Refer to Table S5 for the values of σ = σ_aerogel 1.1 (A1),_ *L* and *A*)

From the calculations above, it can be concluded that *R­_i_* is dominated by the p-type leg, *R_p_*, and the summed contribution of the stretchable Ag used as an n-type leg. The contact resistance is negligible: *R_n_ + R_c_* = 0.2 Ω. Thus, it can be assumed for simplicity that *R_n_*  ≃ *R_c_* ≃ 0. The fact that *R_n_* is negligible compared to *R_p_* is not surprising given the much higher electrical conductivity of the stretchable Ag paste compared to the PEDOT:PSS aerogel 1.1 (A1) (σ_silver_ = 2 10^4^ S/cm Vs. σ_aerogel 1.1 (A1)_= 16 S/cm).

Taking into account the previous considerations, Eq. S11 becomes:

$$P_{max}\approx\frac{\left[ \left( S_{p}-S_{n} \right){\Delta T}_{L} \right]^{2}}{4R_{p}} \left( Eq.S13 \right)$$

To understand the mechanical strain invariability of the output power observed for the stretchable device in Figure 6d, a strain-dependent version of Eq. S13 is proposed:

$$\begin{aligned} P_{max}\left( Ɛ \right)=\frac{\left[ \left( S_{p}(Ɛ)-S_{n}(Ɛ) \right){\Delta T}_{L} \right]^{2}}{4\left( R_{p}\left( Ɛ \right) \right)}\#\left( Eq.S14 \right) \end{aligned}$$

The Seebeck coefficient has been demonstrated to be invariant to the applied strain^[20]^. Hence: *S­_p,n_(ε)* ≃ *S­_p,n_*. The change in electrical resistance of the arched PEDOT:PSS (# 1.1 (A1)) p-type leg is negligible (ΔR/R_0_ < 1.5%) below 10% of applied strain, as shown in Figure 5b. Accordingly, *R_p_(ε)* ≃ *R_p_* and Eq. S14 becomes Eq. S13, demonstrating that the output power is, in practice, strain-independent up to ~ 10% strain, as shown in Figure 6d.

**
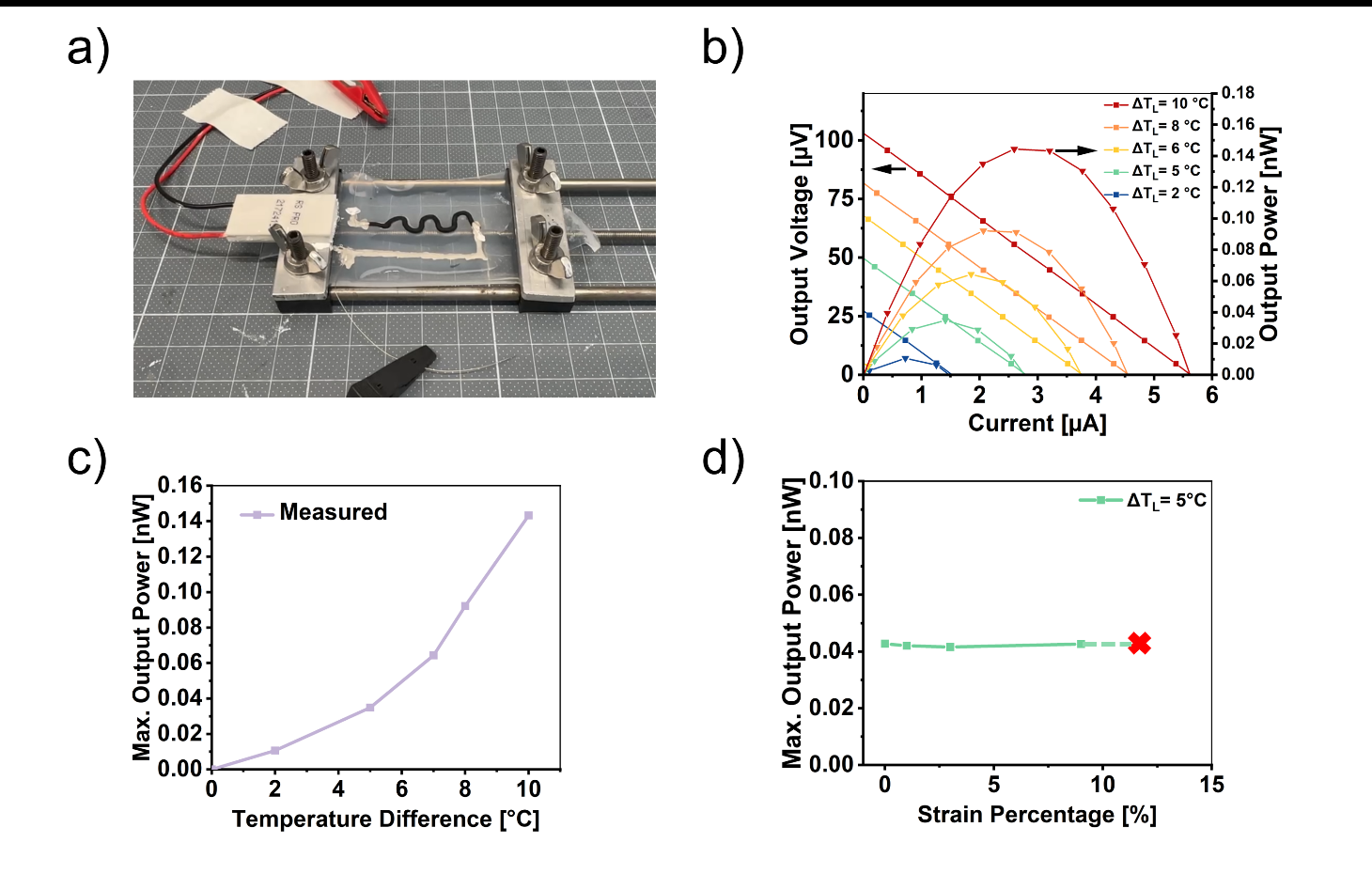
**

**Figure S11. Stretchable planar thermoelectric generators based on in-plane printed serpentine.** a) Optical photograph of a planar stretchable thermocouple composed of in-plane PEDOT: PSS (material composition (A1)) arches and painted stretchable silver. The device is mounted in a house-made stretching set-up and coupled to a Peltier unit at one end to generate a thermal gradient. b) Output voltage and output power vs. output current for different resistive loads and temperature differences across the device. c) Maximum power output at different temperature differences across the device. d) Output power evolution vs. applied strain for a 5°C degree temperature difference across the device.

**8. Thermoelectric output power calculations for the vertical device (aerogel sample # 0f (F) Vs dense sample # 0*)**

To calculate the theoretical maximum output power of the dense (*P_max, dense_*) and aerogel (*P_max, aerogel_*) thermoelectrical pillar legs with a free cold side, a single-leg variation of Eq. S11 can be drawn:

$$P_{max}=\frac{V_{OC}^{2}}{4R_{i}}=\frac{\left[ S{\Delta T}_{L} \right]^{2}}{4\left( R_{L}+R_{c} \right)} \left( Eq.S15 \right)$$

Where the total internal electrical resistance (*R_i_*) of the pillar is the sum of the leg electrical resistance (*R_L_*) and contact electrical resistance (*R_C_*). This is *R_i_* = *R_L_* + *R_C_* according to the electrical circuit model in Figure S12a. Note that the contact electrical resistance *R_C_* is composed of the top (*R_C1_*) and bottom (*R_C2_*) contact resistance: *R_C_* = *R_C1_ + R_C1_*. Using Pouillet’s law (as in Eq. S12) for *R_L_,* we can express *R_i_* as:

$R_{i}=\frac{1}{\sigma}\frac{L}{A}+R_{C} (Eq. S16)$

The pillar leg's thermal resistance (*R'_L_*) can be expressed with the thermal equivalent of Eq. S12 as:

$$\begin{aligned} R_{L}^{'}=\frac{1}{\kappa}\frac{L}{A}\#\left( Eq.S17 \right) \end{aligned}$$

Where κ is the thermal conductivity of the pillar material.

The total heat (*Q*) transferred through the material is, according to Fourier's law for an isotropic medium, given by:

$$\begin{aligned} Q=\frac{{\Delta T}_{E}}{\sum_{i} R_{i}^{'}}=\frac{{\Delta T}_{E}}{R_{H}^{'}\text{+}R_{L}^{'}\text{+}R_{C}^{'}}\#\left( Eq.S18 \right) \end{aligned}$$

Where *R’_H_* and *R’_C_* are the thermal contact resistance at the hot (substrate-pillar interface) and the cold (leg-air interface) side, respectively (see thermal circuit model in Figure S12b). In this analysis, the Thomson effect and the Joule and Peltier heating can be safely neglected (a “back of the envelope” calculation supports this simplification). The conduction and convection heat losses to the air have also been neglected for analytical simplicity. Therefore, the same heat flows through the pillar's hot and cold sides of the pillar. It is worth noting that the assumption of negligible conduction heat to the air holds well for materials whose thermal conductivity is much higher than the air’s. In the case at hand, however, this simplification may introduce a certain error as κ_aerogel_ = 0.065 W m^-1^ K^-1^ is comparable to κ_air_ = 0.026 W m^-1^ K^-1^ (at ambient conditions)^[22]^. This means that caution should be exercised when performing quantitative analysis, but this limitation should take nothing away from the validity of the qualitative analysis intended in this work.


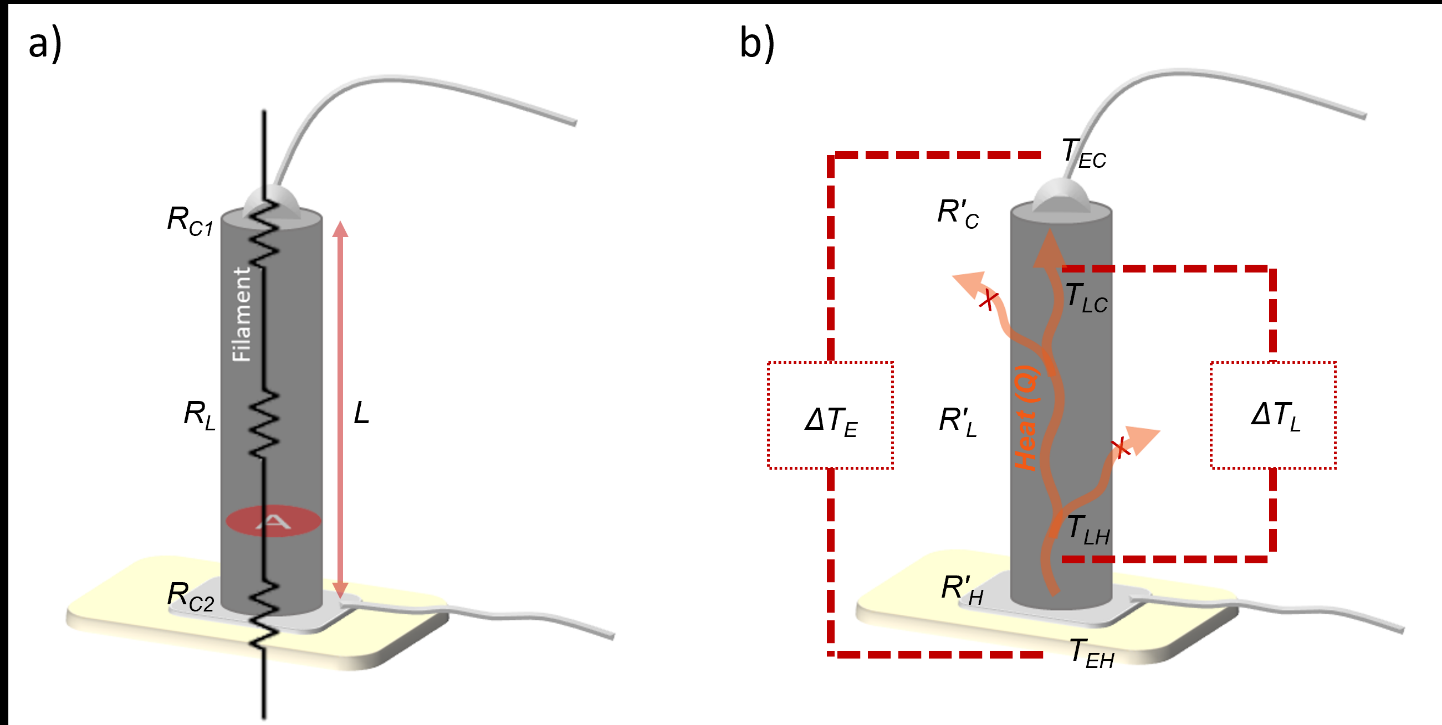


**Figure S12. Vertical pillar legs are deposited on a heated substrate with a natural air-cooled top.** a) Electrical circuit model b) Thermal circuit model**.**

According to Fourier’s law, the drop in temperature across the pillar *ΔT_L_* = *R’_L_* *Q*. Then, using Eq. S18, *ΔT_L_* can be expressed as a function of the externally applied temperature difference (*ΔT_E_*) as:

$$\begin{aligned} {\Delta T}_{L}=R_{L}^{'}Q=\frac{R_{L}^{'}}{R_{H}^{'}\text{+}R_{L}^{'}\text{+}R_{C}^{'}}{\Delta T}_{E}\#\left( Eq.S19 \right) \end{aligned}$$

Substituting in the equation above *R’_L_* for Eq. S17 yields:

$$\begin{aligned} {\Delta T}_{L}=\frac{\frac{1}{\kappa} \frac{L}{A}}{R_{C}^{'}\text{+}R_{H}^{'}\text{+}\frac{1}{\kappa}\frac{L}{A}}{\Delta T}_{E}=\frac{\frac{L}{A}}{\kappa\text{(}R_{C}^{'}\text{+}R_{H}^{'}\text{)}+ \frac{L}{A}}{\Delta T}_{E}\#\left( Eq.S20 \right) \end{aligned}$$

Finally, replacing Eq. S16 and Eq. S20 in Eq. S15, we get an expression for the maximum output power as a function of contact resistances, material properties, and geometry:

$$\begin{aligned} P_{max}= \frac{\left( S {\Delta T}_{E} \right)^{2} \left[ \frac{\frac{L}{A}}{\frac{L}{A}+\kappa\left( R_{H}^{'}+R_{C}^{'} \right)} \right]^{2}}{4\left( \frac{1}{\text{σ}} \frac{L}{A}+R_{C} \right)}\#\left( Eq.S21 \right) \end{aligned}$$

To illustrate the effect of the contact resistances on the performance of TEG, the maximum power output at 300 K is calculated based on Eq.S21 for four different scenarios, accounting for various contact resistances: no contact resistances, only thermal contact resistance, only electrical resistance, and a combination of both thermal and electrical contact resistances. In the absence of any contact resistance, a comparison of an aerogel (like *0f (F)*) with *ZT* ~ 2.4 × 10^-3^ (σ = 13 S/cm and κ = 0.065 W/(m∙K), S=20 μV/K), with a dense material (like 0*) having *ZT* ~ 6 × 10^-3^ (σ = 280 S/cm and κ = 0.55 W/(m∙K), S=20 μV/K) reveals that an aerogel material would produce more than 20 times less power than a dense material: 8 vs. 181 nW (Figure S13a). However, when considering only thermal (Figure S13b) or electrical contact resistance (Figure S13c), the power output difference decreases to less than 3 times or only 1.1 times, respectively. Remarkably, when both contact resistances are taken into account, the aerogel's power output surpasses that of the dense structure by 7 times (0.42 nW vs. 0.06 nW). Unsurprisingly, the max power generated decreases dramatically with both thermal and electrical contact resistances, pointing to the urgent need to find strategies to reduce contact resistance for real applications. However, contact resistance cannot be fully eliminated in real devices, and miniaturization makes the minimization of contact resistance especially challenging.

**
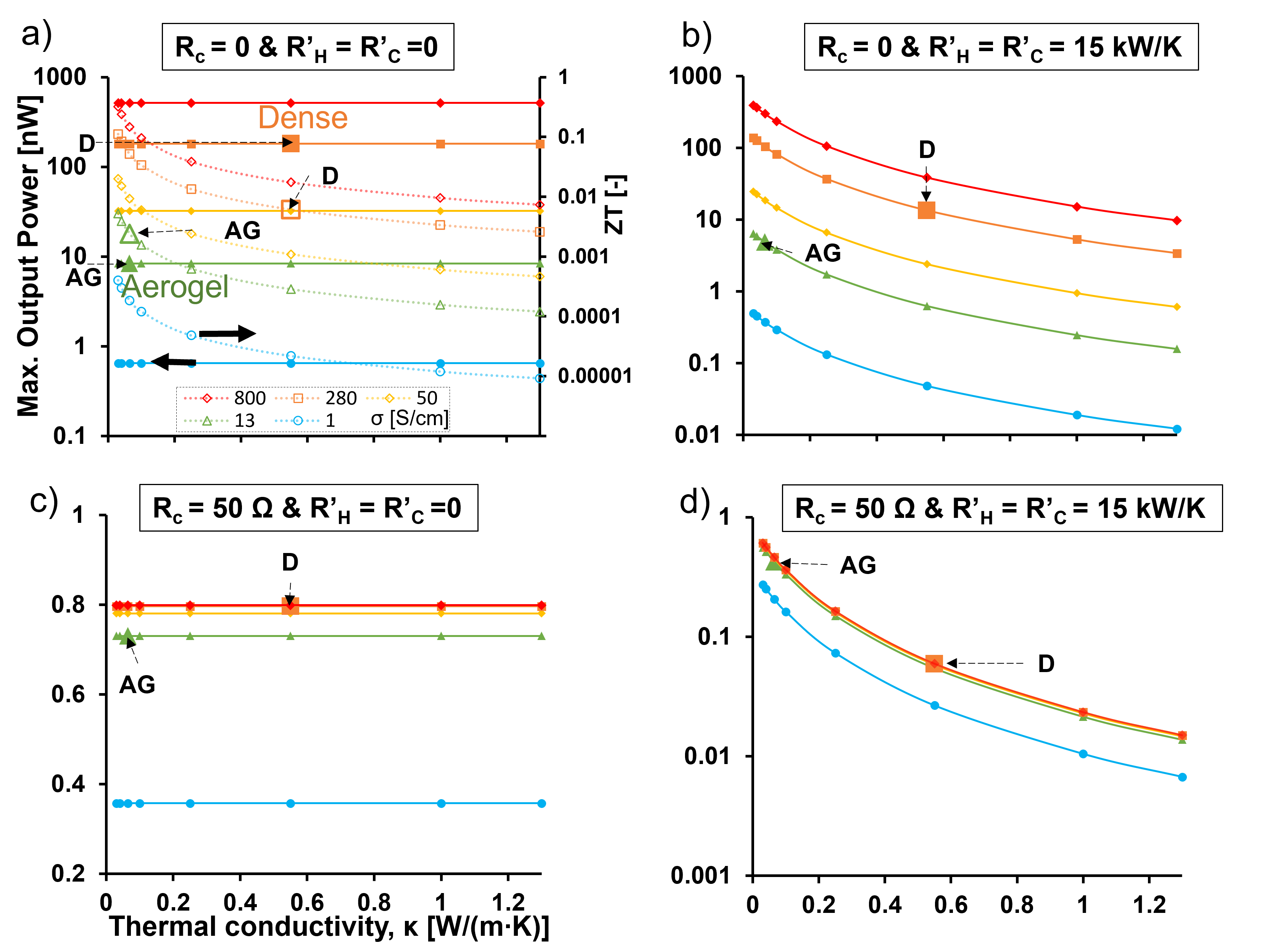
**

**Figure S13. The maximum output power (left y-axis) and the figure of merit (ZT, right y-axis) change for four different cases of electrical and thermal contact resistance.** a) The ideal case: electrical and thermal contact resistances are neglected (R_C_ = 0 & R’_H_ = R’_C_ = 0). b) The thermal contact resistance is taken into account, but the electrical contact resistance is neglected (R_C_ = 0 & R’_H_ = R’_C_ = 15 kW/K). c) The electrical contact resistance is taken into account but the thermal contact resistance is neglected (R_C_ = 50 Ω & R’_H_ = R’_C_ = 0). d) The realistic case: both electrical and thermal contact resistances are taken into account (R_C_ = 50 Ω & R’_H_ = R’_C_ = 15 kW/K). For the figure: S = 20 μV/K, ΔT_E_ = 20 K, L = 3.1 mm and A = 0.5 mm^2^.

**
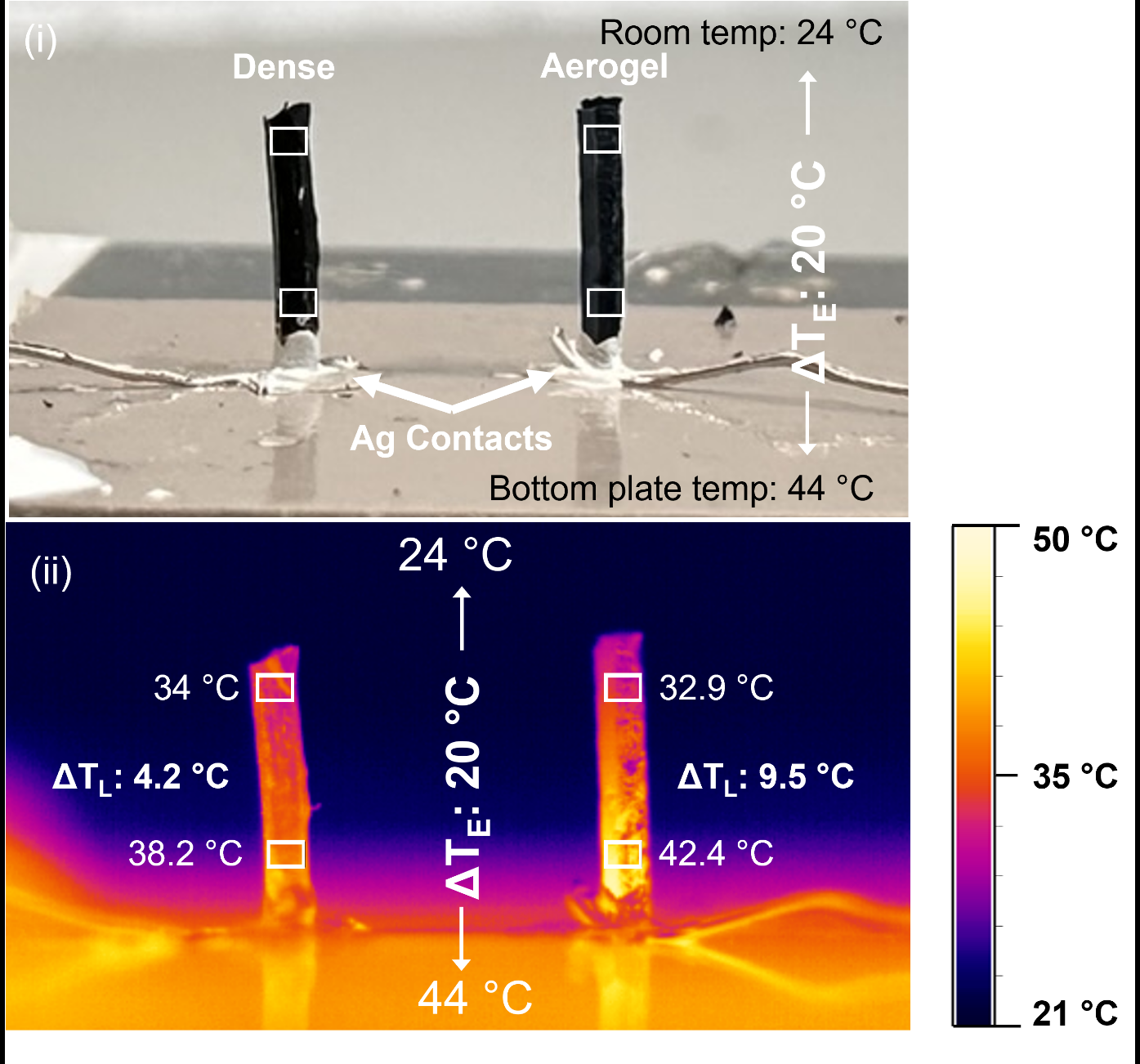
**

**Figure S14. Comparison of the thermal gradient across a dense and a thermoelectric aerogel pillar.** Optical photograph showing the pillars and indication of the external temperature across the device (ΔT_E_) (i), and thermal camera image measurement of the actual temperature difference across the TE leg (ΔT_L_) (ii).

**Table S6. Reported performance of bulky organic thermoelectric materials and through-plane generators.**

| **Study** | **Material** | **Process** | **Structure** | **σ [S/cm]** | **S [μV/K]** | ***PF* [μWm^−1^K^−2^]** | **κ [W/(m K)]** | ***ZT* (x10^-3^)** | **Device Area [mm^2^]** | **Areal Power Density** **[nW/cm^2^]**  **(ΔT=15K)** |
| --- | --- | --- | --- | --- | --- | --- | --- | --- | --- | --- |
| **This Work** | **PEDOT:PSS Aerogel** | **3D Printing** | **Small 3D Pillars** | **13.4 ± 0.3** | **22.7 ± 1.4** | **0.69** | **0.065** | **3.2 ± 0.2** | **0.5** | **26** |
| Ref. ^[23]^ | PEDOT:PSS Aerogel | 3D Printing | 3D Large Objects | 27.15 | 19.34 | 1.02 | 0.075 | 3.99 | 225 | ~0.09 |
| Ref. ^[24]^ | PEDOT:PSS Aerogel | 3D Printing | Small Pillars | 71.9 | - | - | - | - | - | - |
| Ref. ^[25]^ | PEDOT:PSS Aerogel | Casting | Pellet | 7.9 | 20.3 | 0.33 | 0.04 | 2.4 | - | - |
| Ref. ^[26]^ | PEDOT:PSS Aerogel | Casting | Pellet | 2.23 | 17.9 | 0.07 | 0.03 | 0.66 | - | - |
| Ref. ^[27]^ | PEDOT:PSS Aerogel | Casting | Pellet | ~0.1 | - | - | - | - | - | - |
| Ref. ^[19]^ | PEDOT:PSS Aerogel | Casting | Pellet | 18 ± 1 | 18 ± 1 | 0.59 | 0.065 ± 0.003 | 2.7 ± 0.2 | 132 | ~180 |
| Ref.^[28]^ | PEDOT:PSS Aerogel + MgSo4 | Casting | Pellet | 2.9 | 19.9 | 0.11 | 0.053 | 0.62 | - | 90 |
| Ref.^[29]^ | PEDOT:PSS Aerogel | Casting (directional freezing) | Pellet | 12 ± 2 | - | - | - | - | - | - |
| Ref. ^[16]^ | PEDOT: PSS Aerogel | Casting + dipping | Pellet | ~70 | 18 | ~2.3 | - | - | - | - |
| Ref. ^[14]^ | PEDOT:PSS +NMP Compressed Aerogel | Casting and Compressing | Aerogel film | 35 | 18.8 | 1.24 | 0.14 | 2.7 | - | - |
| Ref. ^[30]^ | PEDOT:PSS + NFC + GOPS Aerogel | Casting | Pellet | 0.001 | 37 | 0.14 | - | - | - | - |
| Ref. ^[31]^ | F4TCNQ doped - P3HT foam | Casting | Pellet | 0.22 | 68.4 | 0.103 | 0.14 | 0.23 | - | - |
| **This Work** | **PEDOT:PSS Dense** | **3D Printing** | **Small Pillars (shrank)** | **281 ± 23** | **17.5 ± 0.4** | **8.6 ± 0.7** | **0.55*** | **4.6 ± 0.4** | **0.5** | **24** |
| Ref. ^[32]^ | PEDOT:PSS  Dense | Printing in a Cavity | 3D low aspect ratio pillars | ~1 | ~11 | 0.01 | 0.5 | 0.007 | - | ~1 |
| Ref. ^[33]^ | CNT-PS | Printing in a Cavity | 3D low aspect ratio pillars | 2.1 | 57 | ~6 | - | - | - | ~5.5 |
| Ref.^[34]^ | PEDOT-Tos | Spin-coating | Thin film | ~70 | ~220 | 324 | 0.37 | 250 | - | - |
| Ref. ^[35]^ | PEDOT:PSS – CNT Yarn | Knitting | Fabric | - | - | - | - | - | 3600 | 0.007 |
| Ref. ^[36]^ | PEDOT:PSS Yarn | Knitting | Fabric | 43 ± 10 | 14.3 ± 0.7 | 0.88 ± 0.22 | 0.47 | 0.6 ± 0.1 | 2809 | ~0.017 |

*Owing to the isotropic structure of the bulk pillar, the thermal conductivity value was assumed to be the average of the values reported in the in-plane and through-plane directions for films composed of PEDOT:PSS mixed with DMSO^[4]^.

**References**

[1] H. E. Baysal, F. Molina‐Lopez, in Proc. 2023 IEEE Int. Flex. Electron. Technol. Conf. (IFETC), San Jose, CA, USA, 2023, pp. 1–3.

[2] K. A. Borup, J. De Boor, H. Wang, F. Drymiotis, F. Gascoin, X. Shi, L. Chen, M. I. Fedorov, E. Müller, B. B. Iversen, G. J. Snyder, *Energy Environ. Sci.* 2015, *8*, 423.

[3] I. Petsagkourakis, S. Riera-Galindo, T. P. Ruoko, X. Strakosas, E. Pavlopoulou, X. Liu, S. Braun, R. Kroon, N. Kim, S. Lienemann, V. Gueskine, G. Hadziioannou, M. Berggren, M. Fahlman, S. Fabiano, K. Tybrandt, X. Crispin, *Adv. Sci.* 2023, *10*, 2206954.

[4] Q. Wei, M. Mukaida, K. Kirihara, T. Ishida, *ACS Macro Lett.* 2014, *3*, 948.

[5] J. Liu, X. Wang, D. Li, N. E. Coates, R. A. Segalman, D. G. Cahill, *Macromolecules* 2015, *48*, 585.

[6] A. Krige, J. Haluška, U. Rova, P. Christakopoulos, *HardwareX* 2021, *9*, e00186.

[7] S. Xu, M. Li, M. Hong, L. Yang, Q. Sun, S. Sun, W. Lyu, M. Dargusch, J. Zou, Z. G. Chen, *J. Mater. Sci. Technol.* 2022, *124*, 252.

[8] X. Fan, W. Nie, H. Tsai, N. Wang, H. Huang, Y. Cheng, R. Wen, L. Ma, F. Yan, Y. Xia, *Adv. Sci.* 2019, *6*, 1900813.

[9] Z. Fan, J. Ouyang, *Adv. Electron. Mater.* 2019, *5*, 1800769.

[10] S. Mahato, J. Puigdollers, C. Voz, M. Mukhopadhyay, M. Mukherjee, S. Hazra, *Appl. Surf. Sci.* 2020, *499*, 143967.

[11] D. Alemu Mengistie, P. C. Wang, C. W. Chu, *J. Mater. Chem. A* 2013, *1*, 9907.

[12] G. F. Wang, X. M. Tao, J. H. Xin, B. Fei, *Nanoscale Res. Lett.* 2009, *4*, 613.

[13] J. Park, J. G. Jang, K. Kang, S. H. Kim, J. Kwak, *Adv. Sci.* 2024, *11*, 2308368.

[14] X. Wang, P. Liu, Q. Jiang, W. Zhou, J. Xu, J. Liu, Y. Jia, X. Duan, Y. Liu, Y. Du, F. Jiang, *ACS Appl. Mater. Interfaces* 2019, *11*, 2408.

[15] Y. Wang, C. Zhu, R. Pfattner, H. Yan, L. Jin, S. Chen, F. Molina-Lopez, F. Lissel, J. Liu, N. I. Rabiah, Z. Chen, J. W. Chung, C. Linder, M. F. Toney, B. Murmann, Z. Bao, *Sci. Adv.* 2017, *3*, e1602076.

[16] M. P. Gordon, E. W. Zaia, P. Zhou, B. Russ, N. E. Coates, A. Sahu, J. J. Urban, *J. Appl. Polym. Sci.* 2017, *134*, 44070.

[17] N. F. Uvarov, *Solid State Ionics* 2000, *136*–*137*, 1267.

[18] Q. Weinbach, C. B. Nielsen, L. Biniek, 2021, 10173.

[19] Q. Weinbach, S. V. Thakkar, A. Carvalho, G. Chaplais, J. Combet, D. Constantin, N. Stein, D. Collin, L. Biniek, *Front. Electron. Mater.* 2022, *2*, 875856.

[20] Y. Tian, I. Florenciano, H. Xia, Q. Li, H. E. Baysal, D. Zhu, E. Ramunni, S. Meyers, T. Y. Yu, K. Baert, T. Hauffman, S. Nider, B. Göksel, F. Molina-Lopez, *Adv. Mater.* 2024, *36*, 2307945.

[21] M. Mallick, L. Franke, A. G. Rösch, U. Lemmer, *ACS Energy Lett.* 2021, *6*, 85.

[22] K. Kadoya, N. Matsunaga, A. Nagashima, *J. Phys. Chem. Ref. Data* 1985, *14*, 947.

[23] T. Li, Y. Huang, J. X. M. Chen, Y. C. Sun, O. Aghababaei, Z. Saadatnia, H. E. Naguib, *Nano Energy* 2023, *117*, 108909.

[24] W. Xing, J. Wang, Q. Qian, C. Wang, H. Guo, W. Tan, J. Wu, H. Tang, H. Qi, H. Lin, *ACS Appl. Mater. Interfaces* 2023, *15*, 57717.

[25] N. Yanagishima, S. Kanehashi, H. Saito, K. Ogino, T. Shimomura, *Polymer (Guildf).* 2020, *206*, 122912.

[26] S. Han, F. Jiao, Z. U. Khan, J. Edberg, S. Fabiano, X. Crispin, *Adv. Funct. Mater.* 2017, *27*, 1703549.

[27] X. Zhang, D. Chang, J. Liu, Y. Luo, *J. Mater. Chem.* 2010, *20*, 5080.

[28] J. S. Yun, S. H. Im, *J. Mater. Chem. A* 2024, 7837.

[29] J. Feng, Z. Zhuang, Y. Zhou, C. Li, *Adv. Funct. Mater.* 2024, *34*, 2315188.

[30] Z. U. Khan, J. Edberg, M. M. Hamedi, R. Gabrielsson, H. Granberg, L. Wågberg, I. Engquist, M. Berggren, X. Crispin, *Adv. Mater.* 2016, *28*, 4556.

[31] R. Kroon, J. D. Ryan, D. Kiefer, L. Yu, J. Hynynen, E. Olsson, C. Müller, *Adv. Funct. Mater.* 2017, *27*, 1704183.

[32] M. Massetti, S. Bonfadini, D. Nava, M. Butti, L. Criante, G. Lanzani, L. Qiu, J. C. Hummelen, J. Liu, L. J. A. Koster, M. Caironi, *Nano Energy* 2020, *75*, 104983.

[33] K. Suemori, S. Hoshino, T. Kamata, *Appl. Phys. Lett.* 2013, *103*, 153902.

[34] O. Bubnova, Z. U. Khan, A. Malti, S. Braun, M. Fahlman, M. Berggren, X. Crispin, *Nat. Mater.* 2011, *10*, 429.

[35] Q. Wu, J. Hu, *Smart Mater. Struct.* 2017, *26*, 045037.

[36] A. Lund, Y. Tian, S. Darabi, C. Müller, *J. Power Sources* 2020, *480*, 228836.
